# Design, Synthesis, and Structure–Activity Optimization of Marine-Inspired Macrolactones as Nanomolar Anticancer Agents

**DOI:** 10.1021/acsmedchemlett.6c00222

**Published:** 2026-05-16

**Authors:** Shan Qian, Haibo Qiu, Phillip R. Sanchez, Sarah A. Head, Ruth Hartke, Jun O. Liu, Wei Zheng, Zhendong Jin

**Affiliations:** † Department of Pharmaceutical Sciences and Experimental Therapeutics, College of Pharmacy, 4083 The University of Iowa, Iowa City, Iowa 52242, United States; ‡ Department of Pharmacology and Molecular Sciences, 1500 Johns Hopkins University School of Medicine, Baltimore, Maryland 21205, United States; § National Center for Advancing Translational Sciences (NCATS), 2511 National Institutes of Health, Rockville, Maryland 20892, United States

**Keywords:** Superstolide A analogue, ZJ-101 analogue, anticancer agent, drug design, macrolide

## Abstract

Inspired by the anticancer marine natural product superstolide A, we previously designed and synthesized a truncated analogue, designated ZJ-101, that retains the potent anticancer activity of the original natural product. In this study, using ZJ-101 as a lead compound, we conducted molecular design, convergent synthesis, and structure–activity optimization, resulting in the discovery of new analogues exhibiting anticancer activity approximately 30–80-fold more potent than ZJ-101, with IC_50_ values predominantly in the single-digit nanomolar range.

## Graphical abstract

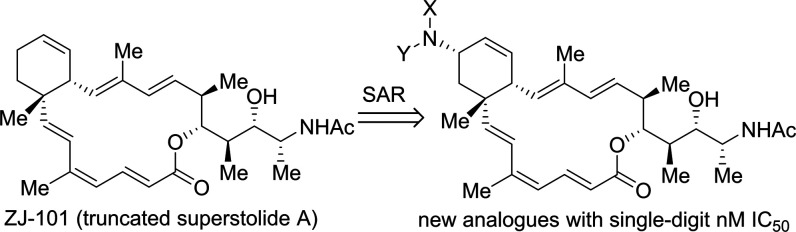

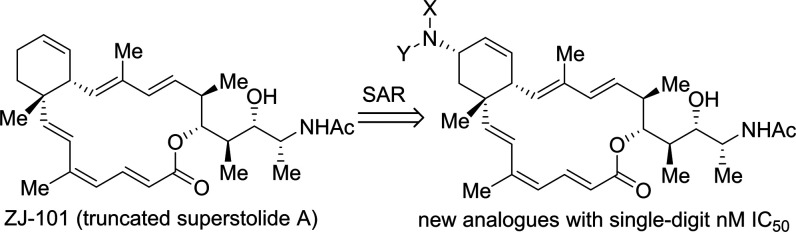

The deep-water marine sponge *Neosiphonia superstes* is a fascinating marine organism localized in the region of New Caledonia in the South Pacific.^1^ Over the years, it has been recognized as a rich source of marine natural products with novel chemical structures and unique biological activities.^2^ Prof. Luigi Minale’s team successfully isolated a number of marine anticancer natural products, including superstolides and sphinxlides from this sponge, and subsequently determined their chemical structures.^3^ However, the development of such marine-derived leads is often limited by scarce compound supply due to the difficulty and environmental impact of deep-sea collection, as well as the challenges of sponge aquaculture.^4^ Moreover, the structural complexity of these natural products renders total synthesis impractical, creating a major bottleneck in advancing them as drug candidates.

An innovative strategy to address this limitation is the design and synthesis of structurally simplified analogues by removing nonessential portions of the natural product while preserving the core pharmacophore. Such truncated analogues are typically more accessible synthetically and can provide sufficient material for biological evaluation. Nevertheless, structural simplification frequently results in reduced potency, highlighting the importance of careful design.

A few years ago, we applied this strategy to the marine natural product superstolide A (Figure 1). A truncated superstolide A (named ZJ-101) was designed and synthesized.^5^ Importantly, we showed that ZJ-101 retained the potent anticancer activity associated with superstolide A. Our highly convergent and scalable synthesis effectively solved the supply problem of superstolide A, albeit indirectly, and enabled the preparation of more than 250 mg of ZJ-101.^5^

**Figure 1:**
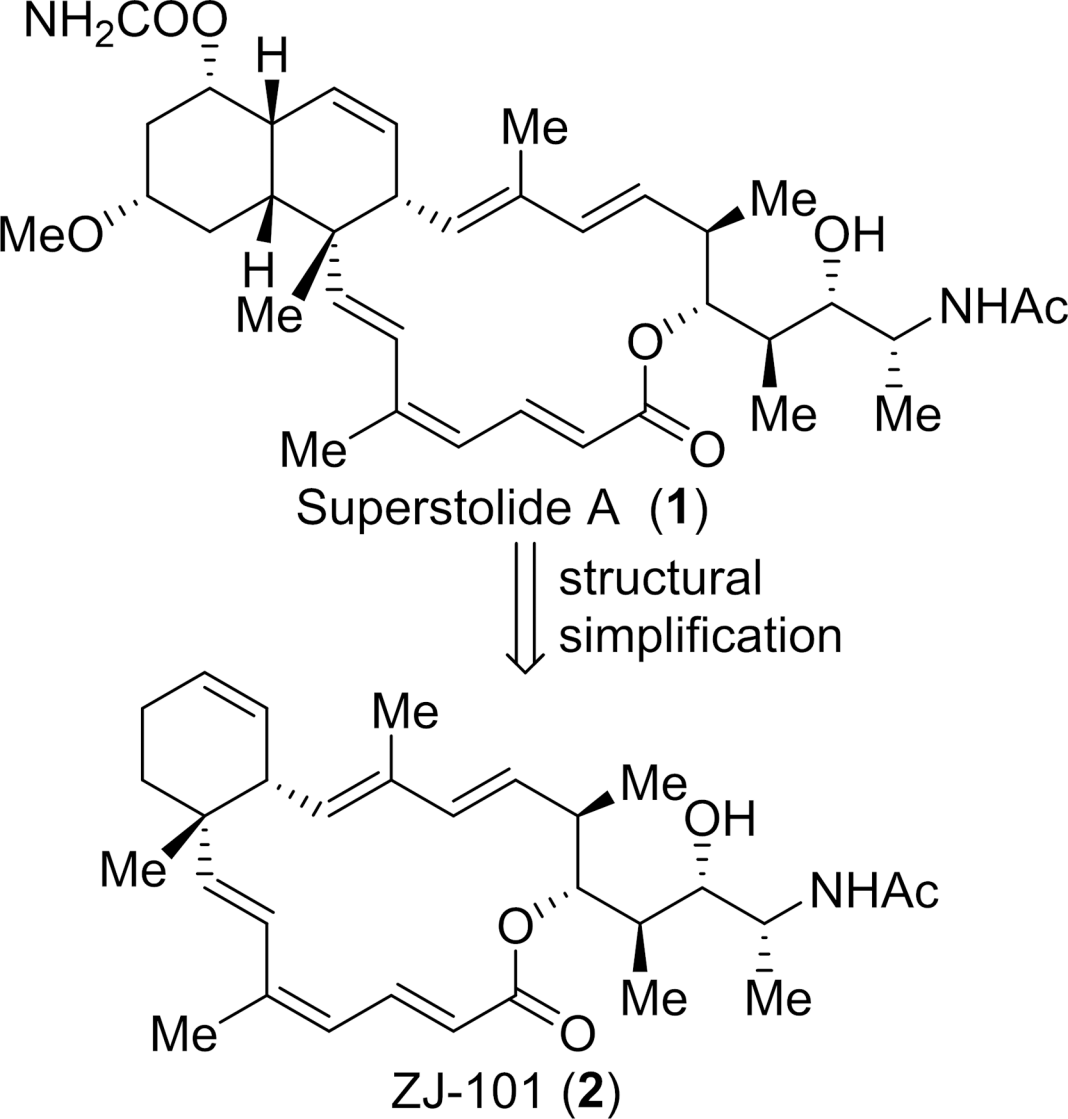
Superstolide A and ZJ-101.

The COMPARE pattern-recognition analysis of ZJ-101’s activity profile against the NCI 60-cell mean graph did not reveal any strong correlations with the profiles of known anticancer compounds in the NCI’s database, suggesting ZJ-101 represents a new class of anticancer agents with a novel and undefined mechanism of action. Our pharmacological study showed that ZJ-101 modulates the endomembrane system by disrupting O-linker glycosylation, a critical process for protein modification and cell adhesion occurring primarily in the Golgi apparatus.^6^ This disruption leads to a significant antiadhesive phenotype, causing cytostatic effect. Notably, the antiadhesion and cytostatic effects of ZJ-101 can synergize with other chemotherapy drugs such as etoposide, enhancing their efficacy and positioning ZJ-101 as a promising candidate for cancer therapy.

The IC_50_ values of ZJ-101 against most human cancer cell lines fall within the range of double-digit nanomolar concentrations.^5^ To develop it into a drug candidate, it is imperative to optimize its potency and ADMET properties. Herein, we report the design, synthesis, and biological evaluation of a panel of ZJ-101-based analogues aimed at optimizing potency.

A significant challenge in optimizing potency arises from the unknown drug target for ZJ-101, rendering structure-based drug design impossible. In addition, the chemical structure of ZJ-101 remains complex. Consequently, synthesizing and screening hundreds of designed analogues with such structural complexity is challenging in an academic setting due to limited resources. To optimize its potency with only a few analogues, careful rational design is required, relying solely on classic medicinal chemistry principles and limited structure–activity relationship (SAR) data from our previous studies.

Our previous SAR study has shown further structural simplification of ZJ-101 results in loss of activity.^7^ To improve ZJ-101’s potency by more than 10-fold, we likely need to increase its binding affinity to the putative receptor. Achieving this requires increasing the structural complexity of ZJ-101. Specifically, we need to stereoselectively introduce at least one potential hydrogen-bonding group at a suitable position. Since we have identified both the conjugated trienyl lactone region of the macrolactone and the acetamide moiety as key pharmacophores,^8^ the cyclohexene ring becomes the logical site for introducing a new hydrogen-bonding group. Among six potential carbons on the ring, it is synthetically easier to modify two allylic positions. Between these, the secondary carbon is more favorable, as a hydrogen-bonding group here would likely enhance receptor binding, whereas modifying the tertiary carbon would create a hindered quaternary center, likely impairing interaction with the target protein(s).

At this allylic position, introducing an alcohol, ester, or ether group often leads to poor metabolic stability and pharmacokinetics because these groups can undergo facile metabolic reactions. Among hydrogen-bonding groups, the amino group offers several distinct advantages: (1) it can be easily converted into various functional groups; (2) it can form water-soluble salts, thereby improving solubility; (3) it can be conjugated to biotin for probe synthesis in target identification; and (4) it can be linked to a monoclonal antibody via a linker for the synthesis of antibody-drug conjugates (ADCs). Therefore, compound **3** was designed (Figure 2). The *S*-configuration of the amino group was chosen solely for its ease of synthesis using our existing strategy. The *R*-isomer of compound **3** will be synthesized if the antiproliferative activity of the *S*-isomer does not meet expectations.

**Figure 2:**
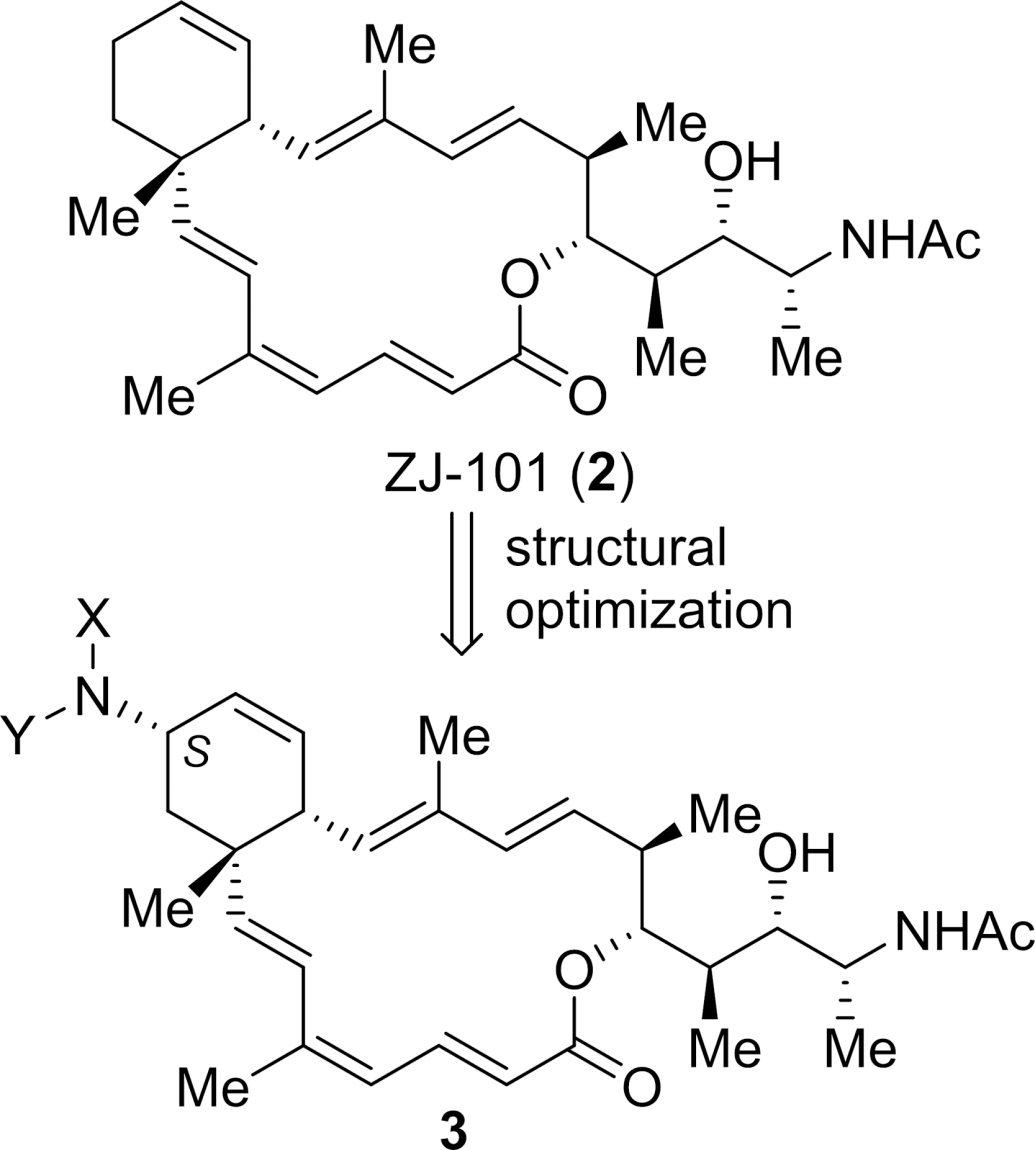
Compound **3**, a new designed analogue of ZJ-101.

The retrosynthetic analysis of compound **3** is outlined in Scheme 1. Compound **3** is derived from its Boc-protected precursor **4**. Sequential disconnections of compound **4** reveal fragments **5**, **6**, and **7** as key intermediates, with Suzuki, Negishi, and Stille couplings playing crucial roles in the synthetic strategy. Compound **5** can be obtained from compound **9** via the intermediate **8**. The overall synthetic strategy for compound **3** is very similar to that of ZJ-101; therefore, there is no need to develop a completely new approach. Moreover, if needed, the *R*-isomer of compound **3** can be synthesized using this strategy with minor modifications.

**Scheme 1:**
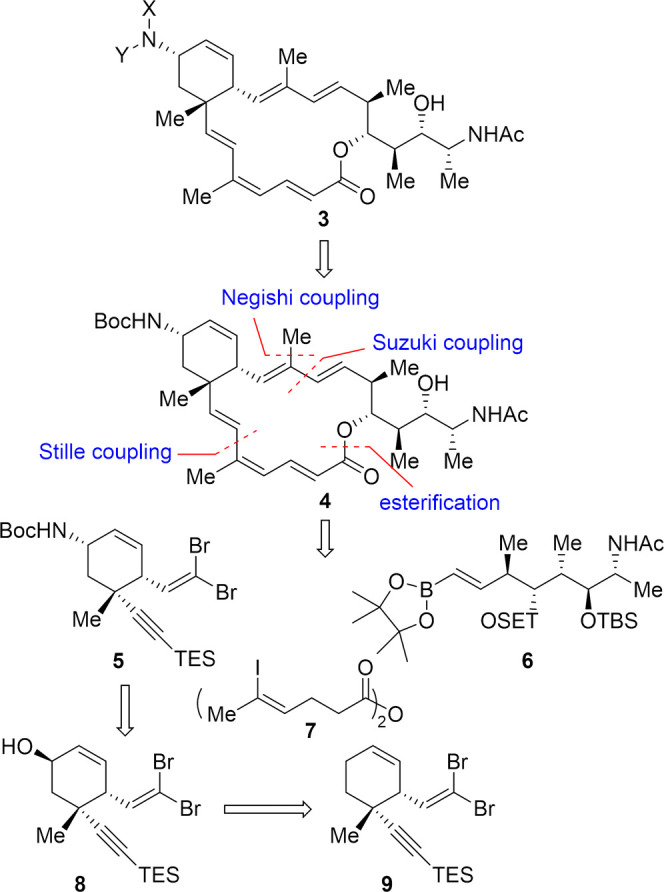
Retrosynthetic Analysis of Compound **3**

The synthesis of fragment **5** is outlined in Scheme 2. Compound **9**, an advanced intermediate in the synthesis of ZJ-101,^5^ reacted with SeO_2_ to give allylic alcohol **8** in 35% yield (55% BRSM). Compound **8** underwent a Mitsunobu reaction to provide azide **10** with the requisite stereochemistry. Staudinger reduction followed by Boc protection yielded compound **5** in 74% yield.

**Scheme 2:**
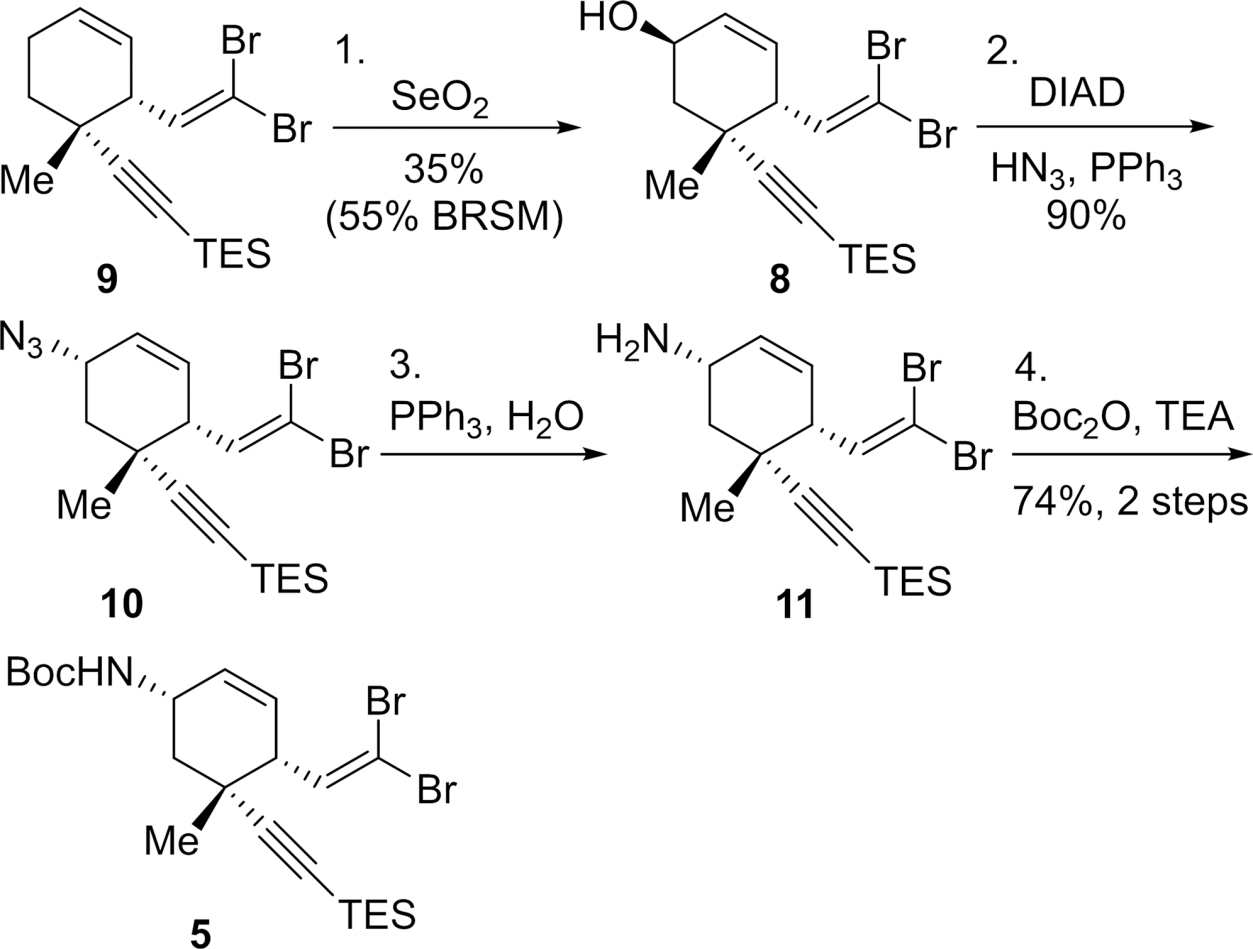
Synthesis of Fragment **5** ^a^

a Reagents and conditions: (1) SeO_2_, dioxane, 80 °C, (35%, 55% BRSM); (2) DIAD, HN_3_, PPh_3_, THF, 25 °C (90%); (3) PPh_3_, H_2_O, 80 °C; (4) Boc_2_O, NEt_3_, DCM, 25 °C (74%, 2 steps).

Anhydride **7** was prepared from its corresponding carboxylic acid **12**, a compound used in our previous synthesis of ZJ-101,^5^ under standard reaction conditions (Scheme 3).

**Scheme 3:**
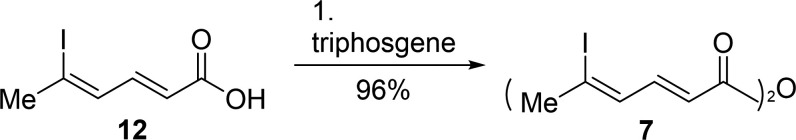
Synthesis of Anhydride **7** ^a^

a Reagents and conditions: (1) triphosgene, NEt_3_, EtOAc, 0–10 °C (96%).

Compounds **5** and **6** underwent Suzuki coupling to give compound **13** in 50% yield (Scheme 4). Negishi coupling between compound **13** and dimethyl zinc provided compound **14** in 73% yield. TBAF-mediated deprotection of three silyl protecting groups in compound **14** yielded compound **15** in 93% yield. Hydrostannylation of terminal alkyne **15** gave compound **16** in 98% yield with complete regioselectivity and stereoselectivity. Esterification between compounds **16** and **7** provided compound **17**, which underwent an intramolecular acyl transfer in the presence of titanium tetraisopropoxide to give compound **18** in 95% yield. Intramolecular Stille coupling then afforded compound **4** in 70% yield.

**Scheme 4:**
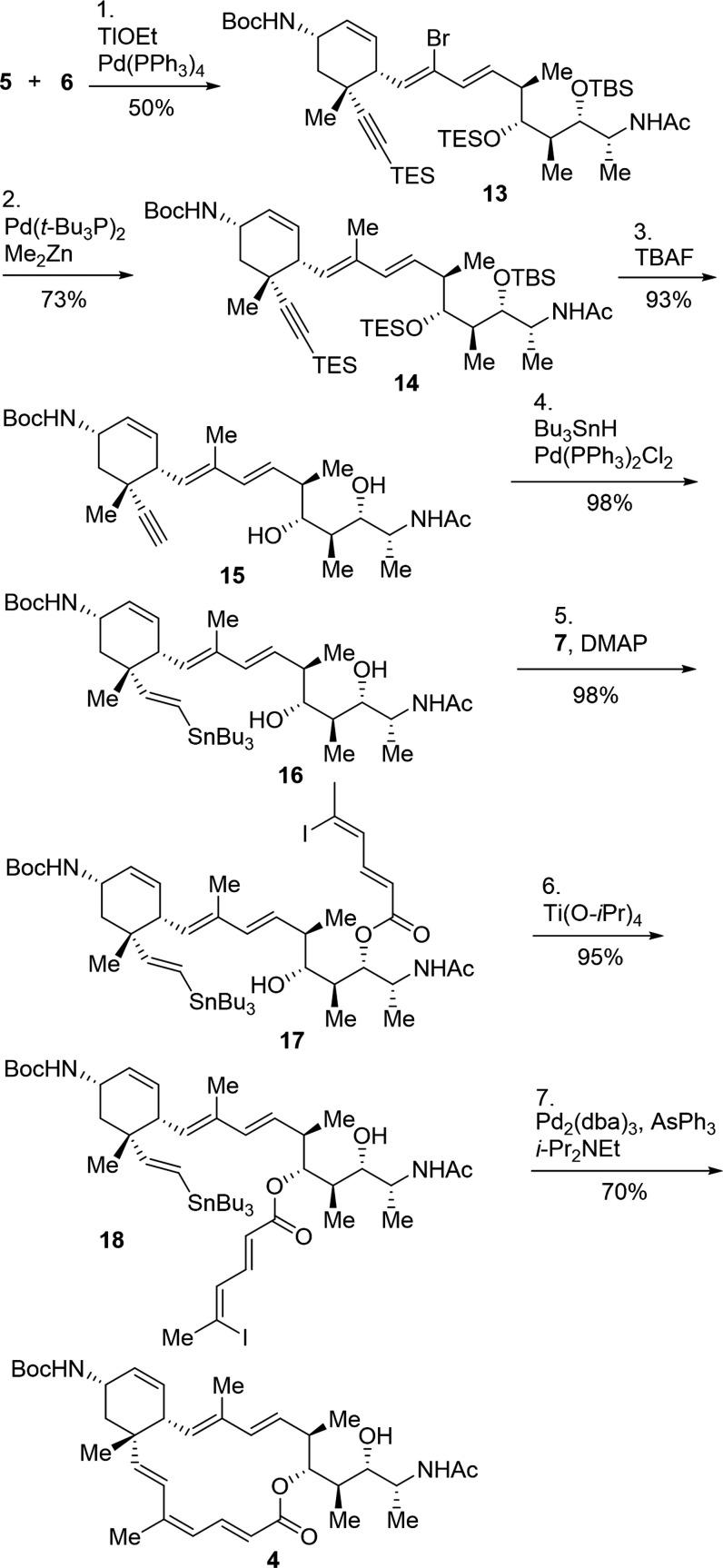
Synthesis of Compound **4** ^a^

a Reagents and conditions: (1) Pd­(PPh_3_)_4_, TlOEt, degassed THF/H_2_O, 25 °C (50%); (2) Pd­(*t*-Bu_3_P)_2_, ZnMe_2_, 0 °C (73%); (3) TBAF, THF, 0 °C and then 25 °C (93%); (4) Bu_3_SnH, Pd­(PPh_3_)_2_Cl_2_, THF, 25 °C, THF/hexanes (1/7) (98%); (5) anhydride **7**, DMAP, THF/toluene (1:7), 0 °C and then 25 °C (98%); (6) Ti­(*i*-PrO)_4_, DCM, 25 °C (95%); (7) Pd_2_(dba)_3_, AsPh_3_, *i*-Pr_2_NEt, DMF (0.001 M), 25 °C (70%).

The antiproliferative effects of compound **4** were evaluated in SF-295, MCF-7, MDA-MB-231, HCT-116, RKO, and HeLa cell lines using the Alamar Blue viability assay, with ZJ-101 as the positive control (Table 1).^9^ Notably, compared with ZJ-101, compound **4** was four times more potent in the RKO cell line and twice as potent in the other five cell lines, suggesting that the stereoselective introduction of Boc-protected amino group at the specific allylic position increased potency. The successful design, synthesis and biological testing of compound **4** paved the way for further structural optimization.

**Table 1:** Antiproliferative Effect (IC_50_ in nM) of ZJ-101 Analogues on Various Malignant Tumor Cells (Alamar Blue Assay)^a^

Cell Line	ZJ-101	**4**	**3a**	**3b**	**3c**	**3d**	**3e**	**3f**	**3g**	**3h**
SF-295	63.48	34.63	15.44	2.22	4.35	NT^a^	NT^a^	NT^a^	NT^a^	NT^a^
MCF-7	40.82	30.09	6.28	1.56	2.66	1.53	3.38	3.71	11.61	6.81
MDA-MB-231	61.15	34.78	9.99	2.23	3.34	1.79	5.06	4.81	12.85	4.43
HCT-116	88.19	31.64	NT^a^	1.33	2.64	1.53	3.64	3.10	12.48	6.58
RKO	192.88	46.86	8.80	2.46	3.57	NT^a^	NT^a^	NT^a^	NT^a^	NT^a^
HeLa	75.44	27.66	7.46	1.80	2.73	NT^a^	NT^a^	NT^a^	NT^a^	NT^a^
293T	159.40	NT^a^	15.17	4.13	5.59	2.92	5.83	7.07	36.16	17.51
A549	33.51	NT^a^	5.33	1.22	1.78	1.39	3.36	3.29	8.14	NT^a^

a “NT” means the compound was not tested in that assay.

Removal of the Boc protecting group in compound **4** yielded a primary amine **3a**, which was easily converted compounds **3b**, **3c**, **3d**, **3e**, **3f**, **3g**, and **3h** in good yields using standard reaction conditions (Scheme 5).

**Scheme 5:**
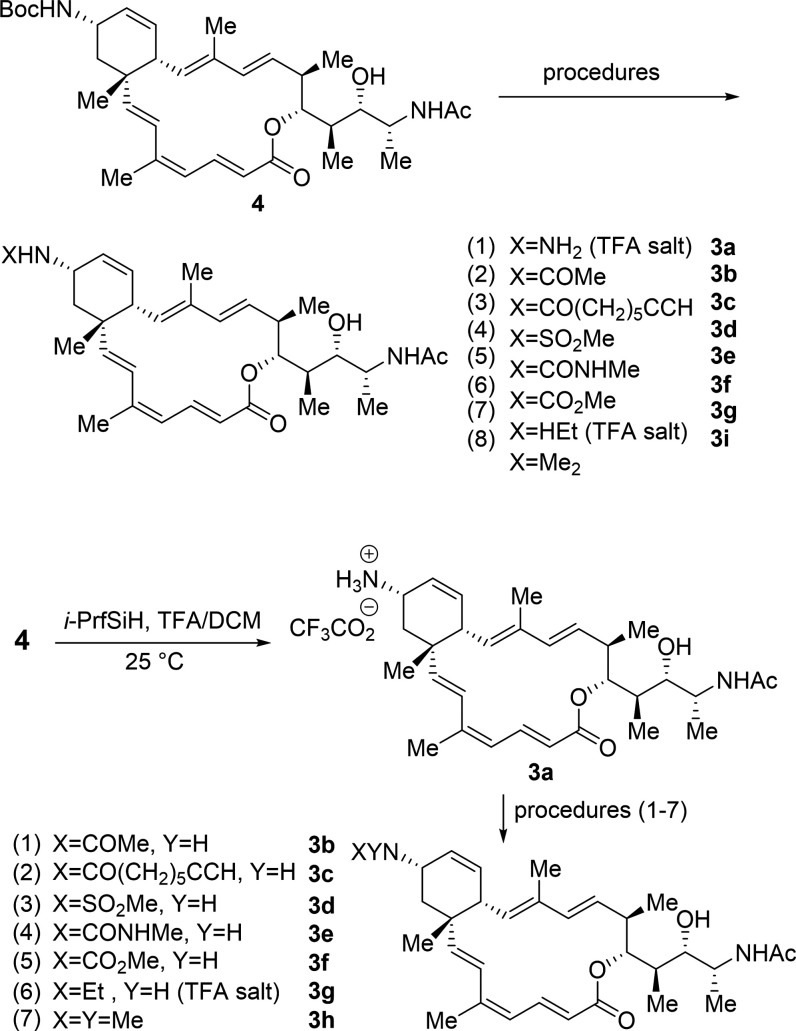
Synthesis of Designed Analogues

a Reagents and conditions: (1) *N*-acetoxysuccinimide, NEt_3_, DCM, 25 °C (85%). (2) 2,5-dioxopyrrolidin-1-yl oct-7-ynoate, NEt_3_, DCM, 25 °C (68%). (3) MeSO_2_Cl, NEt_3_, DCM, 0–25 °C (80% for 2 steps). (4) *N*-methyl-1*H*-imidazole-1-carboxamide, NEt_3_, DCM, 0–25 °C (89% for 2 steps). (5) methyl chloroformate, NEt_3_, DCM, 0–25 °C (59% for 2 steps). (6) (i) MeCHO, MgSO_4_, MeOH; (ii) NaBH_3_CN; (iii) Boc_2_O, THF/aq. NaHCO_3_ (1:1); (iv) TFA/DCM (10/1), *i*-Pr_3_SiH, 25 °C (70% for 5 steps). (7) HCHO, NaBH_3_CN, AcOH, MeCN, 25 °C (80% for 2 steps).

The antiproliferative effects of these eight analogues were determined using various human cancer cell lines in the Alamar Blue viability assay with ZJ-101 as the positive control (Table 1). These eight compounds are all much more potent than ZJ-101. It is particularly exciting to discover that five analogues, **3b** through **3f**, each containing a different amide bioisostere group, exhibit similar potency, with IC_50_ values all in the single-digit nM range. Among them, compound **3b** is the most potent. Although three amine analogues (**3a**, **3g**, and **3h**) are slightly less potent than five analogues (**3b** through **3f**), they are still much more potent than ZJ-101. The biological testing data confirm our design hypothesis that the stereoselective introduction of a new hydrogen-bonding group at the allylic position of the cyclohexene ring increases the compound’s binding affinity for the putative receptor.

In conclusion, we have designed and successfully synthesized eight analogues of ZJ-101. These compounds are about 30–80 times more potent that ZJ-101, with IC_50_ values predominantly in single-digit nanomolar. These results validated our original design hypothesis that introducing an amino moiety at the allylic position of the cyclohexene ring was a sound idea. These compounds are currently undergoing further testing and preclinical evaluation. It should be noted that two amine analogues (**3a** and **3g**) can be used for the synthesis of molecular probes for target identification, and have the potential to be used as novel payloads in antibody-drug conjugates (ADCs) for the treatment of cancer. Research in these directions are currently underway and will be reported in due course.

**Safety Statement.** No unexpected or unusually high safety hazards were encountered during the experiments.

## Supplementary Material



## Notes

Detailed experimental procedures, characterization data, and spectra for new compounds, and biological assay protocol (PDF)

## ABBREVIATIONS

DCM: dichloromethane
DIAD: diisopropyl azodicarboxylate
DMAP: 4-dimethylaminopyridine
DMF: dimethylformamide
DMSO: dimethyl sulfoxide
Pd_2_(dba)_3_: tris­(dibenzylideneacetone)­dipalladium (0)
TBAF: tetra-*n*-butylammonium fluoride
TBS: *tert*-butyldimethylsilyl
TES: triethylsilyl
TFA: trifluoroacetic acid
