# Supplementary material for: Design, Synthesis, and Structure–Activity Optimization of Marine-Inspired Macrolactones as Nanomolar Anticancer Agents
Source: ACS Med Chem Lett. 2026 May 16;17(6):1416–20. doi: 10.1021/acsmedchemlett.6c00222 (PMC13266650; doi:10.1021/acsmedchemlett.6c00222)
Supplement: Supplementary file 1 [file ml6c00222_si_001.pdf]

Supporting Information

Design, Synthesis, and Structure-Activity Optimization of Marine-Inspired Macrolactones as Nanomolar Anticancer Agents

*Shan Qian[a],[†] Haibo Qiu[a],[†] Phillip R. Sanchez[b], Sarah A. Head[b], Ruth Hartke[b], Jun O. Liu[b], Wei Zheng[c], and Zhendong Jin[a]**

a. Department of Pharmaceutical Sciences and Experimental Therapeutics, College of Pharmacy, The University of Iowa, Iowa City, IA 52242, USA

b. Department of Pharmacology and Molecular Sciences, Johns Hopkins University School of Medicine, Baltimore, MD 21205 USA

c. National Center for Advancing Translational Sciences (NCATS), National Institutes of Health, Rockville, MD 20892, USA

***Corresponding author**: Zhendong Jin, Email: zhendong-jin@uiowa.edu

Table of Contents

General Methods 3
Experimental Procedures and Data 4
Abbreviations 15
NMR Spectra 16

General Methods

Unless stated otherwise, reactions were performed in flame-dried glassware under a positive pressure of argon using freshly distilled solvent. Tetrahydrofuran (THF) and diethyl ether were distilled from sodium/benzophenone before use. Dichloromethane and toluene were distilled from CaH_2. Anhydrous methanol (99.8%) was bought from Aldrich. Thin-layer chromatography (TLC) was performed using Dynamic Adsorbents silica gel w/h F-254 250 μm glass plates. Visualization of the developed chromatography was performed by UV absorbance (254 nm) and visualizing solutions. The commonly employed TLC visualizing stains were: anisaldehyde solution and 12-molybdophosphoric acid solution. Column chromatography was performed using Dynamic absorbents silica gel (32-63 μm).

All ^{1}H-NMR and ^{13}C-NMR spectra were recorded with a Bruker Advance300 (300 MHz). In reported ^{1}H NMR spectra, data are presented as follows: chemical shift (in ppm on the δ scale relative to δ H 7.26 for the residual protons in $CDCl_3$ and δ H 7.16 for the residual protons in C_6D_6), integration, multiplicity (*s* = singlet, *d* = doublet, *t* = triplet, *q* = quartet, *m* = multiplet, *br* = broad), coupling constant (*J*/Hz). Coupling constants were taken directly from the spectra and are uncorrected. In reported ^{13}C NMR spectra, all chemical shift values are reported in ppm on the δ scale, with an internal reference of δ C 77.16 for $CDCl_3$ and δ C 128.06 for C_6D_6. Mass spectral determinations were carried out by using electrospray ionization as ionization source (ESI). Optical rotations were measured on Jasco P-1020 polarimeters. Melting points are uncorrected.

Experimental Procedures and data

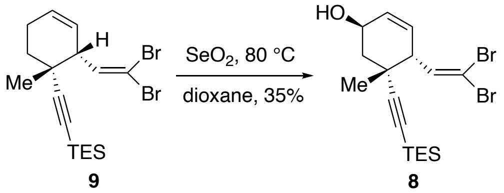

To a stirred solution of dibromo alkene **9** (330 mg, 0.789 mmol) in dioxane (40.0 mL) at 80 ℃ was added SeO_2 (350 mg, 3.156 mmol) in one portion. The resulting mixture was stirred at that temperature for 12 hours. After cooling to room temperature, the mixture was filtered through a pad of celite. The filtrate was concentrated in vacuum to dryness and the residue was purified by column chromatography (R_f = 0.60, hexane/ethyl acetate = 3/1) on silica gel to give the allylic alcohol **8** (120 mg, 35%) and recovered starting material **1** (132 mg, 40%).

$[\alpha]^{21}_D$ = +48.7 (*c* 1.00, $CHCl_3$); ^{1}H NMR (300 MHz, $CDCl_3$): δ 6.59 (d, *J* = 9.9 Hz, 1H), 5.82 (d, *J* = 10.8 Hz, 1H), 5.38 (dt, *J* = 9.9, 1.8 Hz, 1H), 4.57 (brs, 1H), 2.95 (dq, *J* = 9.9, 2.7 Hz, 1H), 2.23 (ddd, *J* = 12.3, 5.7, 1.2 Hz, 1H), 1.49 (brs, 1H), 1.43 (dd, *J* = 12.3, 10.2 Hz, 1H), 1.27 (s, 3H), 0.96 (t, *J* = 7.8 Hz, 9H), 0.55 (q, *J* = 7.8 Hz, 6H); ^{13}C NMR (75 MHz, $CDCl_3$): δ 138.8, 132.0, 127.1, 109.9, 90.5, 84.6, 66.5, 50.5, 44.6, 36.7, 28.3, 7.5, 4.5 HRMS (ESI): calcd. for $C_{17}H_{26}Br_2NaOSi$ $[M+Na]^+$: 455.0017, found: 455.0030.

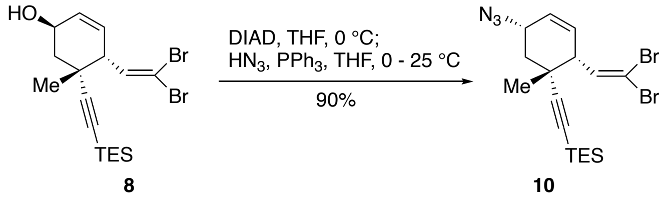

At 0 ℃ a solution of DIAD (3.47 g, 16.32 mmol) in THF (10.0 mL) was added into another solution of alcohol **8** (2.20 g, 5.10 mmol), HN_3 in toluene solution (6.38 mL, 12.75 mmol) and PPh_3 (4.01 g, 15.3 mmol) in THF (26.0 mL). After completion of the addition, the resulting mixture was stirred at room temperature until alcohol **8** disappeared on the TLC. Concentration of the mixture provided a residue, which was purified by column chromatography (R_f = 0.50, hexane/ethyl acetate = 20/1) on silica gel and afforded the allylic azide **10** (2.11 g, 90%).

$[\alpha]^{20}_D$ = +26.7 (*c* 1.00, $CHCl_3$); ^{1}H NMR (300 MHz, $CDCl_3$): δ 6.49 (d, *J* = 9.6 Hz, 1H), 5.80 (dt, *J* = 9.6, 2.4 Hz, 1H), 5.72 (dq, *J* = 11.2, 1.5 Hz, 1H), 3.98 – 3.89 (m, 1H), 2.97 – 2.89 (m, 1H), 1.94 (dd, *J* = 6.0, 2.7 Hz, 2H), 1.30 (s, 3H), 0.99 (t, *J* = 7.8 Hz, 9H), 0.58 (q, *J* = 7.8 Hz, 6H); ^{13}C NMR (75 MHz, $CDCl_3$): δ 138.0, 129.7, 124.9, 110.4, 90.5, 84.8, 53.7, 49.2, 38.3, 33.0, 27.1, 7.5, 4.4; HRMS (ESI): calcd. for $C_{17}H_{25}Br_2N_3NaSi$ $[M+Na]^+$: 480.0082, found: 480.0070.

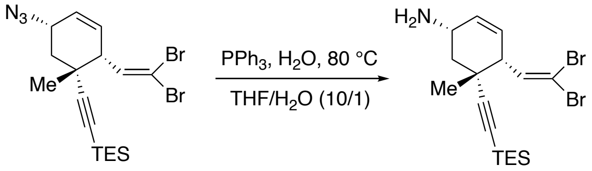

10 **11**

To a stirred solution of azide **10** (2.11 g, 4.59 mmol) in THF (150.0 mL) and H_2O (15.0 mL) at room temperature was added PPh_3 (12.05 g, 45.94 mmol) in one portion. The resulting mixture was stirred at 80 ℃ until no starting material was observed. After cooling to room temperature, the mixture was concentrated, and toluene was added to remove H_2O azeotropically. The crude product **11** was used directly for the next step without further purification.

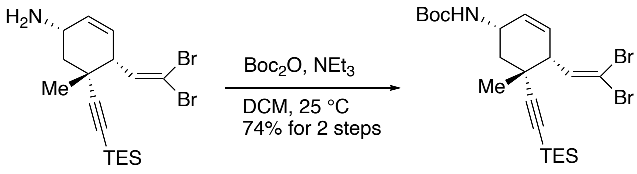

11 **5**

To a stirred solution of amine **11** in DCM (150.0 mL) at room temperature was added NEt_3 (6.4 mL) followed by Boc_2O (2.0 g, 9.18 mmol). The resulting mixture was stirred at room temperature until the reaction was complete. Concentration of the mixture provided a residue, which was purified by column chromatography (R_f = 0.60, hexane/ethyl acetate = 10/1) on silica gel to give provided compound **5** (1.80 g, 74% for 2 steps).

$[\alpha]^{18}_{D}$ = +12.0 (*c* 1.00, $CHCl_3$); ^1H NMR (300 MHz, $CDCl_3$): δ 6.58 (d, *J* = 9.9 Hz, 1H), 5.86 – 5.77 (m, 1H), 5.72 (d, *J* = 10.2 Hz, 1H), 5.46 (dt, *J* = 10.2, 1.8 Hz, 1H), 4.35 – 4.24 (m, 1H), 2.83 (dq, *J* = 9.9, 2.4 Hz, 1H), 2.02 (d, *J* = 14.1 Hz, 1H), 1.82 (dd, *J* = 14.1, 6.3 Hz, 1H), 1.43 (s, 9H), 1.25 (s, 3H), 1.00 (t, *J* = 8.1 Hz, 9H), 0.60 (q, *J* = 8.1 Hz, 6H); ^{13}C NMR (75 MHz, $CDCl_3$): δ 155.1, 138.9, 128.6, 127.59, 112.0, 90.6, 85.8, 79.2, 50.1, 44.10, 4.4, 32.9, 28.4, 28.1, 7.5, 4.3; HRMS (ESI): calcd. for $C_{22}H_{35}Br_2NNaO_2Si$ $[M+Na]^+$: 554.0702, found: 554.0717.

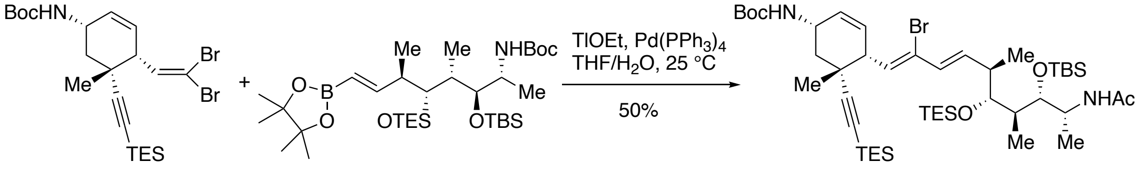

5 **6** **13**

To a stirred solution of dibromoolefin **5** (340 mg, 0.637 mmol) and vinyl boronic ester **6** (521 mg, 0.892 mmol) in degassed THF/H_2O (9.0 mL/3.0 mL) at room temperature was added $Pd(PPh_3)_4$ (73.6 mg, 0.0637 mmol). The mixture was stirred at room temperature for 5 min, and then thalliumethylate (0.18 mL, 2.550 mmol) was added dropwisely. After stirring at room temperature for 0.5 h, the reaction mixture was diluted with EtOAc and H_2O. Then the solid was removed by filtration through a pad of celite. The organic layer was separated, and the aqueous phase was extracted with EtOAc for 3 times. The combined organic layers were washed with brine and dried over anhydrous Na_2SO_4. The filtrate was

concentrated in vacuum and the residue was purified by column chromatography (R_f = 0.50, hexanes/ethyl acetate = 3/1) to yield compound **13** (291 mg, 50%).

$[\alpha]^{19}_D$ = +18.2 (*c* 1.00, $CHCl_3$); ^1H NMR (300 MHz, $CDCl_3$): δ 6.18 – 6.05 (m, 1H), 6.05 (d, *J* = 15.0 Hz, 1H), 5.92 – 5.75 (m, 2H), 5.55 – 5.40 (m, 2H), 5.08 – 4.97 (m, 1H), 4.31 (brs, 1H), 4.08 – 3.95 (m, 1H), 3.74 (dt, *J* = 6.3, 2.4 Hz, 1H), 3.62 (td, *J* = 7.2, 3.0 Hz, 1H), 3.18 (dq, *J* = 9.6, 1.8 Hz, 1H), 2.75 – 2.45 (m, 1H), 2.10 – 2.00 (m, 1H), 1.94 (s, 3H), 1.88 – 1.66 (m, 3H), 1.44 (s, 9H), 1.19 (s, 3H), 1.13 – 1.06 (m, 6H), 1.03 – 0.88 (m, 29H), 0.70 – 0.55 (m, 12H), 0.06 – 0.00 (m, 6H); ^{13}C NMR (75 MHz, $CDCl_3$): δ 168.3, 155.2, 138.0, 132.2, 129.41, 129.3, 127.8, 127.5, 112.9, 85.1, 79.1, 74.6, 48.6, 46.9, 44.3, 42.6, 42.2, 42.0, 41.7, 41.0, 33.1, 28.4, 28.2, 25.9, 23.5, 18.3, 15.5, 11.6, 7.6, 7.2, 5.6, 4.3, -3.9, -4.7; HRMS (ESI): calcd. for $C_{46}H_{85}BrN_2NaO_5Si_3$ $[M+Na]^+$: 931.4847, found: 931.4860.

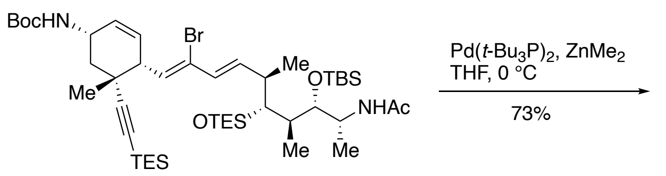

To a solution of $Pd(t\text{-}Bu_3P)_2$ (13.5 mg, 0.0264 mmol) in 2.6 mL degassed THF was added a solution of $ZnMe_2$ (0.79 mL, 2.0 M in toluene, 1.581 mmol) at 0 ℃. This solution was cannulated into a flask of neat compound **13** (480 mg, 0.527 mmol) at 0 ℃. After stirring at 0 ℃ for 5 h, the reaction was quenched with sat. aq. $NaHCO_3$ carefully and diluted with DCM. The organic phase was separated, and the aqueous phase was extracted with DCM for 3 times. The combined organic layers were washed with brine and dried over anhydrous Na_2SO_4. The filtrate was concentrated, and the residue was purified by column chromatography (R_f = 0.50, hexanes/ethyl acetate = 3/1) on silica gel to give compound **14** (325 mg, 73%).

$[\alpha]^{20}_D$ = +13.2 (*c* 1.00, $CHCl_3$); ^1H NMR (300 MHz, $CDCl_3$): δ 6.07 (d, *J* = 15.6 Hz, 1H), 5.84 (d, *J* = 11.5 Hz, 1H), 5.75 – 5.50 (m, 3H), 5.41 (d, *J* = 9.6 Hz, 1H), 5.05 – 4.96 (m, 1H), 4.33 – 4.22 (brs, 1H), 4.01 (t, *J* = 6.9 Hz, 1H), 3.75 – 3.68 (m, 1H), 3.60 (dd, *J* = 6.6, 2.7 Hz, 1H), 2.79 (d, *J* = 9.6 Hz, 1H), 2.58 – 2.46 (m, 1H), 2.18 – 1.97 (m, 1H), 1.91 (s, 3H), 1.86 – 1.66 (m, 2H), 1.72 (s, 3H), 1.42 (s, 9H), 1.23 (d, *J* = 2.4 Hz, 1H), 1.15 – 0.84 (m, 38H), 0.68 – 0.48 (m, 12H), 0.04 – -0.04 (m, 6H); ^{13}C NMR (75 MHz, $CDCl_3$): δ 168.2, 155.2, 135.3, 135.0, 131.1, 130.8, 130.1, 127.2, 113.0, 84.7, 78.9, 74.7, 46.8, 44.7, 42.4, 41.9, 41.6, 33.3, 31.5, 28.4, 28.1, 25.8, 23.4, 22.6, 18.5, 18.2, 15.6, 13.1, 11.5, 7.5, 7.1, 5.6, 4.3, -4.1, -4.8; HRMS (ESI): calcd. for $C_{47}H_{88}N_2NaO_5Si_3$ $[M+Na]^+$: 867.5899, found: 867.5912.

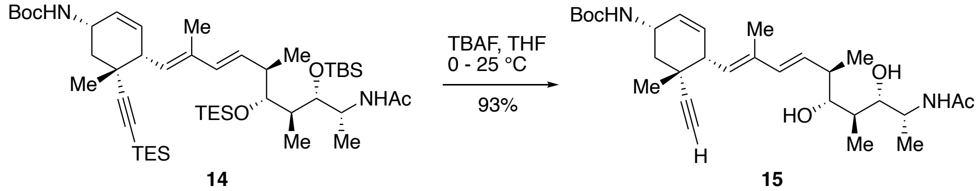

To a stirred solution of compound **14** (337 mg, 0.40 mmol) in 10 mL THF was added a solution of TBAF (2.39 mL, 1.0 M in THF, 2.39 mmol) dropwisely at 0 ℃. The reaction was gradually warmed up to room temperature and stirred overnight (> 12 h). The reaction was quenched with H_2O and diluted with DCM.

The organic phase was separated, and the aqueous phase was extracted with DCM for 3 times. The combined organic layers were washed with brine and dried with anhydrous Na_2SO_4. The filtrate was concentrated, and the residue was purified by column chromatography (R_f = 0.45, DCM/MeOH = 10/1) on silica gel to give alkyne **15** (187 mg, 93%).

$[\alpha]^{19}_D$ = +42.3 (*c* 1.00, $CHCl_3$); ^1H NMR (300 MHz, $CDCl_3$): δ 6.22 (d, *J* = 15.6 Hz, 1H), 6.18 – 6.10 (m, 1H), 5.76 – 5.68 (m, 1H), 5.58 – 5.41 (m, 3H), 4.32 – 4.22 (brs, 1H), 4.18 – 4.06 (m, 1H), 3.76 – 3.61 (m, 2H), 3.52 (dd, *J* = 11.7, 6.0 Hz, 1H), 2.97 – 2.88 (m, 1H), 2.81 (dd, *J* = 8.1, 1.8 Hz, 1H), 2.35 – 2.22 (m, 2H), 2.14 (s, 1H), 2.05 – 1.97 (m, 1H), 1.94 (s, 3H), 1.89 – 1.65 (m, 3H), 1.74 (s, 3H), 1.42 (s, 9H), 1.19 – 1.09 (m, 5H), 0.98 – 0.90 (m, 6H); ^{13}C NMR (75 MHz, $CDCl_3$): δ 169.2, 155.2, 136.8, 134.9, 131.5, 130.8, 130.8, 127.3, 88.9, 79.2, 73.9, 71.5, 47.0, 44.2, 41.5, 35.3, 32.5, 28.4, 27.9, 25.3, 23.5, 20.1, 16.7, 15.0, 13.5, 13.1, 9.7; HRMS (ESI): calcd. for $C_{29}H_{46}N_2NaO_5$ $[M+Na]^+$: 525.3304, found: 525.3289.

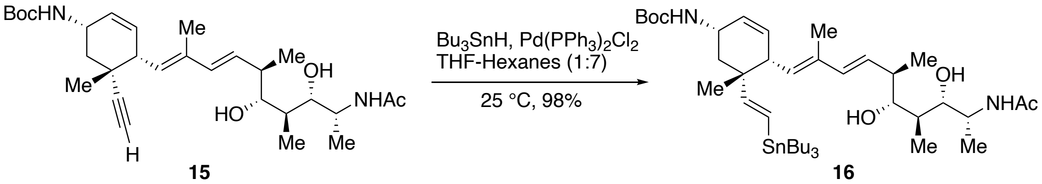

15 16

To a stirred solution of alkyne **15** (26 mg, 0.0517 mmol) in THF (2.0 mL) containing $Pd(PPh_3)_2Cl_2$ (1.8 mg, 0.00259 mmol) at room temperature was added a solution of Bu_3SnH (30 mg, 0.1034 mmol) in 1.0 mL of THF-Hexanes (1:7) over hours via a syringe pump. After the reaction was complete, the reaction mixture was concentrated in vacuum, and the residue was purified by column chromatography (R_f = 0.50, DCM/MeOH = 10/1) on silica gel to give compound **16** (40 mg, 98%).

$[\alpha]^{18}_D$ = +73.2 (*c* 0.50, $CHCl_3$); ^1H NMR (300 MHz, $CDCl_3$): δ 6.11 (d, *J* = 15.6 Hz, 1H), 5.82 (s, 1H), 5.80 – 5.68 (m, 1H), 5.62 – 5.50 (m, 2H), 5.35 (dd, *J* = 15.6, 9.0 Hz, 1H), 5.15 (d, *J* = 9.9 Hz, 1H), 4.57 (d, *J* = 9.0 Hz, 1H), 4.28 – 4.12 (m, 2H), 3.68 (dt, *J* = 9.6, 1.8 Hz, 1H), 3.49 (q, *J* = 6.6 Hz, 1H), 3.25 (d, *J* = 6.6 Hz, 1H), 2.84 – 2.76 (m, 1H), 2.28 – 2.20 (m, 1H), 1.97 (s, 3H), 1.90 – 1.78 (m, 2H), 1.74 (s, 3H), 1.68 – 1.59 (m, 1H), 1.52 – 1.42 (m, 3H), 1.45 (s, 9H), 1.40 – 1.22 (m, 8H), 1.17 (d, *J* = 6.6 Hz, 3H), 1.09 (s, 3H), 0.98 – 0.78 (m, 24H); ^{13}C NMR (75 MHz, $CDCl_3$): δ 169.0, 155.7, 155.4, 137.7, 132.9, 132.5, 130.2, 129.5, 127.5, 123.5, 79.3, 73.9, 47.0, 43.8, 41.8, 41.1, 34.9, 29.0, 28.4, 27.2, 26.8, 25.0, 23.6, 17.5, 16.5, 15.5, 13.7, 13.6, 12.9, 9.9, 9.4; HRMS (ESI): calcd. for $C_{41}H_{74}N_2NaO_5Sn$ $[M+Na]^+$: 817.4517, found: 817.4531.

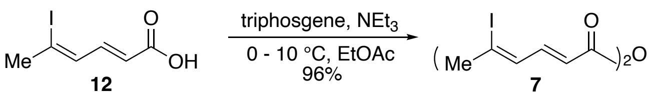

12 7

A solution of acid **12** (401 mg, 1.685 mmol) and NEt_3 (0.24 mL, 1.685 mmol) in EtOAc (13.0 mL) was allowed to react with triphosgene (85 mg, 0.078 mmol) at 0 ℃ for 10 min and at room temperature for additional 0.5 h. The reaction mixture was filtered through a pad of celite, washed with EtOAc, and the filtrate was evaporated to dryness and afforded anhydride **7** (385 mg, 96%).

^{1}H NMR (300 MHz, $CDCl_3$): δ 7.46 (dd, J = 15.3, 10.5 Hz, 1H), 6.32 (dq, J = 10.5, 0.6 Hz, 1H), 6.06 (dt, J = 15.3, 0.6 Hz, 1H), 2.72 (t, J = 0.6 Hz, 3H); ^{13}C NMR (75 MHz, $CDCl_3$): δ 162.2, 149.8, 132.6, 121.7, 115.6, 35.3; HRMS (ESI): calcd. for $C_{12}H_{12}I_2NaO_3$ $[M+Na]^+$: 480.8773, found: 480.8789.

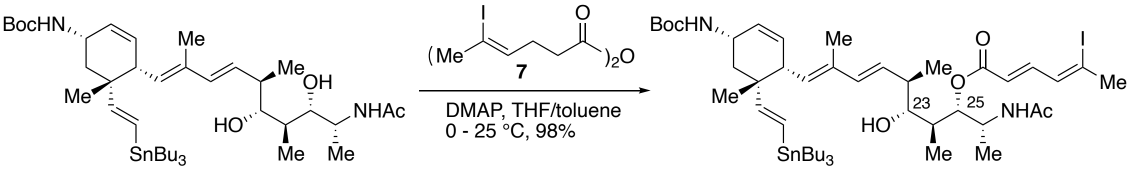

16 17

To a stirred solution of diol **16** (367 mg, 0.462 mmol) and DMAP (113 mg, 0.925 mmol) in THF/toluene (6.0 mL/42.0 mL, v/v = 1/7) at 0 ℃ was added anhydride **7** (233 mg, 0.509 mmol) in 10.0 mL toluene dropwisely over 1 h. The resulting mixture was allowed to warm up to room temperature and stirred until no starting material was observed. Concentration of the mixture in vacuum provided a residue, which was purified by flash column chromatography (R_f = 0.65, DCM/MeOH = 10/1) to give ester **17** (459 mg, C25-ester/C23-ester > 8/1) in 98% combined yield. The crude ester **13** was directly used without further characterization.

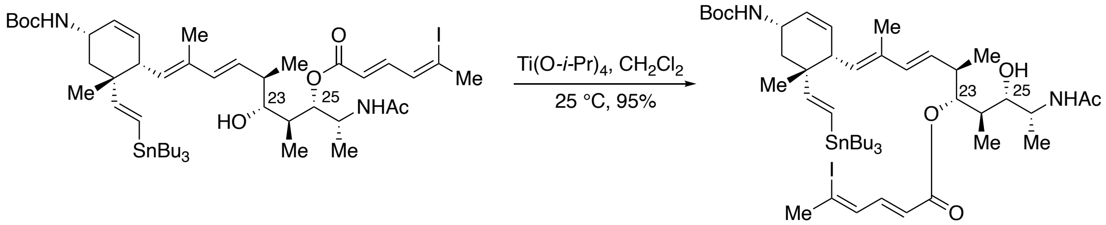

17 18

To a stirred solution of esters **17** (459 mg, 0.453 mmol) in 100 mL DCM was added $Ti(O\text{-}i\text{-}Pr)_4$ (1.34 mL, 4.53 mmol) dropwisely at room temperature. After stirring at room temperature overnight (> 12 h) until no further changes on the TLC plates were observed. Then the reaction was quenched with sat. aq. $NaHCO_3$ and diluted with DCM. The mixture was stirred for 15 min, and then the organic phase was separated. The aqueous phase was extracted with DCM for 3 times. The combined organic layers were washed by brine and dried with anhydrous Na_2SO_4. The filtrate was concentrated, and the residue was purified by column chromatography (R_f = 0.60, DCM/MeOH = 10/1) to give ester **18** (436 mg, C25-ester/C23-ester < 1/10) in 95% combined yield. The crude ester **18** was directly used without further characterization.

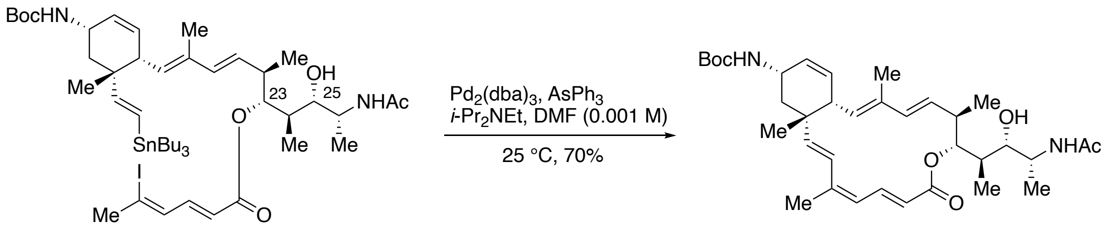

18 4

To a solution of ester **18** (238 mg, 0.235 mmol) in 235 mL DMF was added $i\text{-}Pr_2NEt$ (0.41 mL, 2.348 mmol), $AsPh_3$ (58 mg, 0.188 mmol) and $Pd_2(dba)_3$ (43 mg, 0.0470 mmol). The reaction mixture was

degassed by three freeze-pump-thaw cycles. Then the flask was covered with aluminum foil and the reaction mixture was stirred at room temperature over 36 h. The DMF was removed under high vacuum and the residue was purified by column chromatography (R_f = 0.70, DCM/MeOH = 10/1) on silica gel to afford compound **4** (98 mg, 70%).

$[\alpha]^{19}_D$ = +176.0 (*c* 0.50, $CHCl_3$); ^1H NMR (300 MHz, $CDCl_3$): δ 7.13 (dd, *J* = 15.3, 10.8 Hz, 1H), 6.59 (d, *J* = 16.2 Hz, 1H), 6.27 – 6.18 (m, 2H), 5.91 (d, *J* = 10.8 Hz, 1H), 5.73 – 5.52 (m, 5H), 5.38 – 5.25 (m, 2H), 5.12 – 5.06 (m, 1H), 4.80 (d, *J* = 10.8 Hz, 1H), 4.56 (d, *J* = 4.2 Hz, 1H), 4.50 (d, *J* = 9.0 Hz, 1H), 4.32 – 4.11 (m, 3H), 3.13 (dt, *J* = 10.5, 3.6 Hz, 1H), 2.97 (dd, *J* = 9.6, 4.2 Hz, 1H), 2.78 – 2.65 (m, 1H), 2.00 – 1.68 (m, 2H), 1.95 (s, 3H), 1.89 (s, 3H), 1.76 (s, 3H), 1.45 (s, 9H), 1.20 – 1.10 (m, 1H), 1.05 (t, *J* = 6.3 Hz, 6H), 0.86 (d, *J* = 6.9 Hz, 3H); ^{13}C NMR (75 MHz, $CDCl_3$): δ 169.6, 168.9, 155.4, 143.5, 143.3, 139.3, 136.6, 132.7, 131.6, 130.1, 129.6, 126.5, 125.8, 124.3, 120.4, 79.5, 77.2, 73.0, 69.5, 45.4, 42.2, 40.4, 39.4, 37.5, 29.2, 28.4, 26.4, 23.6, 21.5, 18.0, 12.7, 12.6, 8.8; HRMS (ESI): calcd. for $C_{35}H_{52}N_2NaO_6$ $[M+Na]^+$: 619.3723, found: 619.3742.

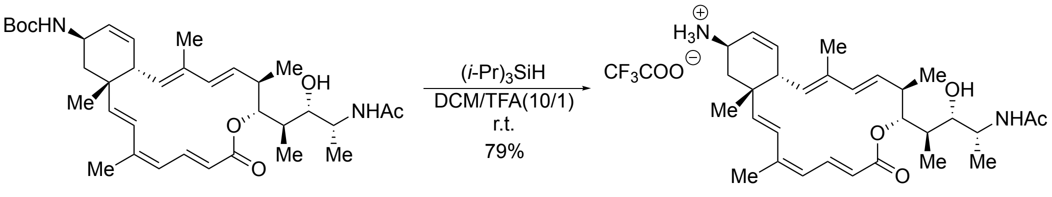

4 **3a**

To a solution of compound **4** (7.1 mg, 0.0119 mmol) in DCM (1.0 mL) and TFA (0.1 mL) was added *i*-Pr_3SiH (7.3 µL, 0.0357 mmol) at room temperature. The resulting mixture was stirred until no starting material was observed. The solvent was removed through filtration under slow nitrogen flow. The crude solid was washed with ethyl ether and dried *in vacuo* to give TFA salt form of compound **3a** as white powder (5.7 mg, 79%).

^1H NMR ($CDCl_3$, 300 MHz): *δ* 8.19 (brs, 3H), 7.12 (dd, *J* = 15.2, 10.3 Hz, 1H), 6.63 (d, *J* = 16.3 Hz, 1H), 6.37 (d, *J* = 8.2 Hz, 1H), 6.23 (d, *J* = 15.1 Hz, 1H), 5.92 (d, *J* = 11.0 Hz, 1H), 5.82 – 5.51 (m, 4H), 5.42 – 5.25 (m, 2H), 4.81 (d, *J* = 10.2 Hz, 1H), 4.13 (brs, 1H), 3.85 (brs, 1H), 3.14 (d, *J* = 10.1 Hz, 1H), 3.08 – 2.98 (m, 1H), 2.76 – 2.59 (m, 1H), 2.00 – 1.79 (m, 4H), 1.96 (s, 3H), 1.87 (s, 3H), 1.75 (s, 3H), 1.13 (s, 3H), 1.10 – 0.97 (m, 6H), 0.84 (d, *J* = 6.3 Hz, 3H).

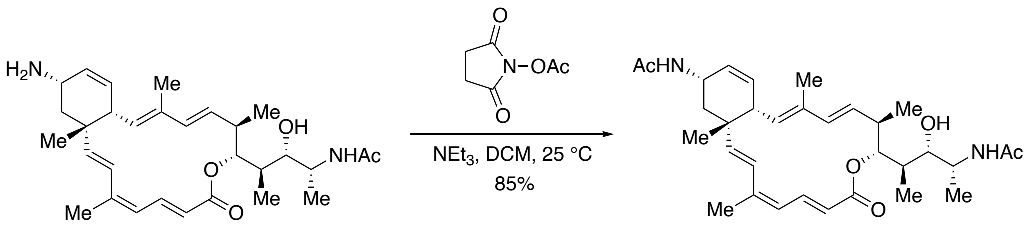

3a **3b**

To a solution of compound **3a** (8.8 mg, 0.0146 mmol) in DCM (2.0 mL) was added NEt_3 (4.1 µL, 0.0293 mmol) and *N*-acetoxysuccinimide (4.6 mg, 0.0293 mmol). The resulting mixture was stirred at room temperature overnight. Concentration of the mixture provided a residue, which was purified by preparative TLC (R_f = 0.35, DCM/MeOH = 15/1) to give compound **3b** as a white solid (6.7 mg, 85%).

$[\alpha]^{19}_{D}$ = +235.9 (*c* 0.28, $CHCl_3$); ^{1}H NMR ($CDCl_3$, 300 MHz): *δ* 7.13 (dd, *J* = 15.2, 11.0 Hz, 1H), 6.60 (d, *J* = 16.4 Hz, 1H), 6.28 – 6.18 (m, 2H), 5.92 (d, *J* = 11.0 Hz, 1H), 5.71 (d, *J* = 11.0 Hz, 1H), 5.65 – 5.49 (m, 3H), 5.42 – 5.25 (m, 3H), 4.81 (d, *J* = 10.4 Hz, 1H), 4.70 – 4.58 (m, 1H), 4.56 (d, *J* = 4.1 Hz, 1H), 4.29 – 4.09 (m, 1H), 3.14 (dt, *J* = 9.7, 3.3 Hz, 1H), 3.00 (dd, *J* = 9.6, 5.0 Hz, 1H), 2.78 – 2.64 (m, 1H), 2.00 (s, 3H), 1.97 (s, 3H), 1.88 (3H s), 1.78 (s, 3H), 1.53 – 1.36 (m, 3H), 1.25 (s, 3H), 1.19 (s, 3H), 1.06 (dd, *J* = 6.5, 5.3 Hz, 3H), 0.87 (d, *J* = 6.9 Hz, 3H); HRMS (ESI, m/z) calcd for $C_{32}H_{46}N_2O_5Na^+$ (M+Na$^+$): 561.3304, found 561.3297.

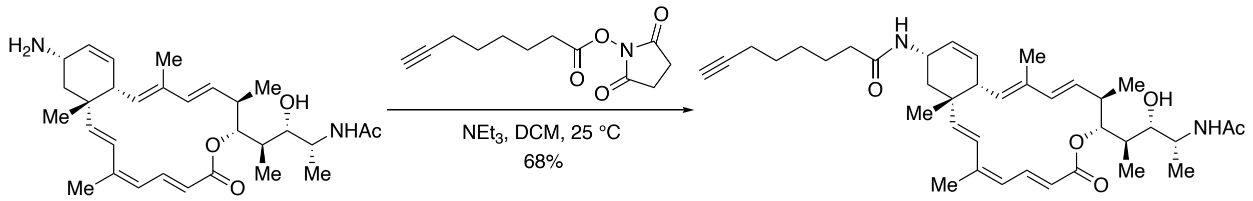

3a **3c**

To a solution of compound **3a** (4.7 mg, 0.00775 mmol) in DCM (1.0 mL) was added NEt_3 (2.2 μL, 0.0155 mmol) and 2,5-dioxopyrrolidin-1-yl oct-7-ynoate (3.7 mg, 0.0155 mmol). The resulting mixture was stirred at room temperature overnight. Concentration of the mixture provided a residue, which was purified by preparative TLC (R_f = 0.35, DCM/MeOH = 15/1) to give compound **3c** as a white solid (3.0 mg, 68%). $[\alpha]^{19}_{D}$ = +156.1 (*c* 0.20, $CHCl_3$); ^{1}H NMR ($CDCl_3$, 300 MHz): *δ* 7.16 (dd, *J* = 15.2, 11.1 Hz, 1H), 6.60 (d, *J* = 16.4 Hz, 1H), 6.30 – 6.16 (m, 2H), 5.92 (d, *J* = 10.7 Hz, 1H), 5.71 (d, *J* = 15.3 Hz, 1H), 5.65 – 5.46 (m, 3H), 5.43 – 5.25 (m, 3H), 4.81 (d, *J* = 10.3 Hz, 1H), 4.71 – 4.58 (m, 1H), 4.56 (d, *J* = 3.7 Hz, 1H), 4.27 – 4.12 (m, 1H), 3.19 – 3.08 (m, 1H), 3.00 (dd, *J* = 9.8, 5.2 Hz, 1H), 2.78 – 2.63 (m, 1H), 2.26 – 2.11 (m, 4H), 1.96 (s, 2H), 1.94 (t, *J* = 2.6 Hz, 1H), 1.88 (s, 3H), 1.78 (s, 3H), 1.73 – 1.61 (m, 3H), 1.60 – 1.52 (m, 3H), 1.53 – 1.36 (m, 4H), 1.25 (s, 3H), 1.19 (s, 3H), 1.06 (dd, *J* = 6.5, 5.4 Hz, 3H), 0.87 (d, *J* = 6.8 Hz, 3H); HRMS (ESI, m/z) calcd for $C_{38}H_{54}N_2O_5Na^+$ (M+Na$^+$): 641.3930, found 641.3941.

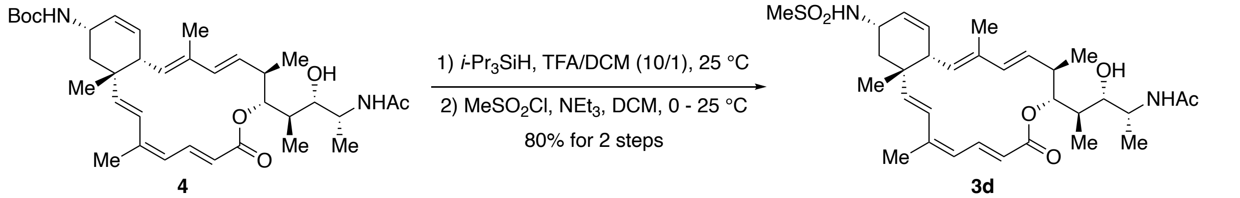

4 **3d**

To a solution of compound **4** (4.4 mg, 0.0074 mmol) in DCM (1.0 mL) and TFA (0.1 mL) was added *i*-Pr$_3$SiH (4.6 μL, 0.0221 mmol) at room temperature. The resulting mixture was stirred until no starting material was observed. The solvent was removed through filtration under slow nitrogen flow. The crude solid was washed with ethyl ether and dried *in vacuo* to give compound **3a** as white powder, which was used for the next step without further purification. To a solution of compound **3a** in DCM (0.5 ml) at 0 °C was added NEt_3 (1.5μL, 0.011 mmol) and $MeSO_2Cl$ (0.74 μL, 0.0096 mmol). After stirring for 0.5 h, the reaction mixture was allowed to warm to room temperature and stirred until the reaction was complete. Saturated aqueous $NaHCO_3$ was added to quench the reaction. The mixture was extracted with DCM three times. Then the combined organic extracts were washed successively with H_2O and brine, dried

over anhydrous Na_2SO_4, filtered and concentrated *in vacuo*. Purification on silica gel column (DCM/MeOH = 50:1 to 15:1) provided compound **3d** (3.4 mg, 80% for two steps) as a white solid.
$[\alpha]^{19}_D$ = +157.9 (*c* 0.11, $CHCl_3$); ^1H NMR ($CDCl_3$, 300 MHz): δ 7.13 (dd, *J* = 15.1, 11.0 Hz, 1H), 6.63 (d, *J* = 16.4 Hz, 1H), 6.33 – 6.14 (m, 2H), 5.93 (d, *J* = 10.7 Hz, 1H), 5.71 (d, *J* = 15.3 Hz, 1H), 5.67 – 5.48 (m, 3H), 5.41 – 5.26 (m, 2H), 4.81 (d, *J* = 10.5 Hz, 1H), 4.55 (d, *J* = 3.9 Hz, 1H), 4.29 – 3.99 (m, 3H), 3.18 – 3.07 (m, 1H), 3.03 (s, 3H), 2.77 – 2.64 (m, 1H), 1.96 (s, 3H), 1.89 (s, 3H), 1.78 (s, 3H), 1.68 – 1.56 (m, 2H), 1.39 – 1.28 (m, 2H), 1.25 (s, 3H), 1.17 (s, 3H), 1.06 (dd, *J* = 6.3, 5.4 Hz, 3H), 0.87 (d, *J* = 6.9 Hz, 3H); HRMS (ESI, m/z) calcd for $C_{31}H_{47}N_2O_6S^+$ (M+H$^+$): 575.3155, found 575.3155.

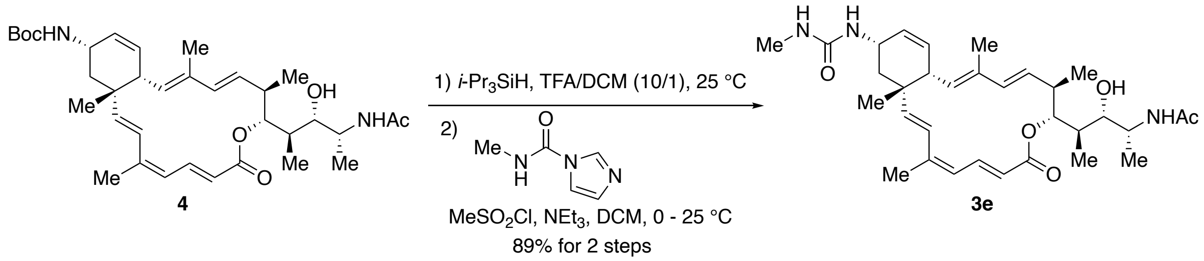

To a solution of compound **4** (4.0 mg, 0.0067 mmol) in DCM (1.0 mL) and TFA (0.1 mL) was added *i*-Pr_3SiH (4.2 µL, 0.0201 mmol) at room temperature. The resulting mixture was stirred until no starting material was observed. The solvent was removed through filtration under slow nitrogen flow. The crude solid was washed with ethyl ether and dried *in vacuo* to get compound **3a** as white powder, which was used for the next step without further purification. To a solution of compound **3a** in DCM (0.5 ml) at 0 °C was added *N*-methyl-1*H*-imidazole-1-carboxamide (1.3 mg, 0.010 mmol) and NEt_3 (1.9 µL, 0.0134 mmol). The reaction mixture was allowed to warm to room temperature and stirred until the reaction was complete. The mixture was concentrated *in vacuo*. Purification on silica gel column (DCM/MeOH = 50:1 to 10:1) provided compound **3e** (3.2 mg, 89% for two steps) as a white solid.
$[\alpha]^{19}_D$ = +157.7 (*c* 0.22, MeOH); ^1H NMR ($CDCl_3$, 300 MHz): δ 7.13 (dd, *J* = 15.2, 11.0 Hz, 1H), 6.59 (d, *J* = 16.5 Hz, 1H), 6.33 – 6.15 (m, 2H), 5.91 (d, *J* = 11.0 Hz, 1H), 5.70 (d, *J* = 15.2 Hz, 1H), 5.65 – 5.48 (m, 3H), 5.42 – 5.26 (m, 2H), 4.80 (d, *J* = 10.6 Hz, 1H), 4.49 – 4.36 (m, 1H), 4.38 (brs, 1H), 4.25 – 4.08 (m, 1H), 3.20 – 3.05 (m, 1H), 3.03 – 2.93 (m, 1H), 2.79 (s, 3H), 2.73 – 2.62 (m, 1H), 1.96 (s, 3H), 1.88 (s, 3H), 1.77 (s, 3H), 1.53 – 1.40 (m, 2H), 1.25 (s, 3H), 1.18 (s, 3H), 1.10 – 1.01 (m, 6H), 0.86 (d, *J* = 6.8 Hz, 3H); HRMS (ESI, m/z) calcd for $C_{32}H_{48}N_3O_5^+$ (M+H$^+$): 554.3594, found 554.3593.

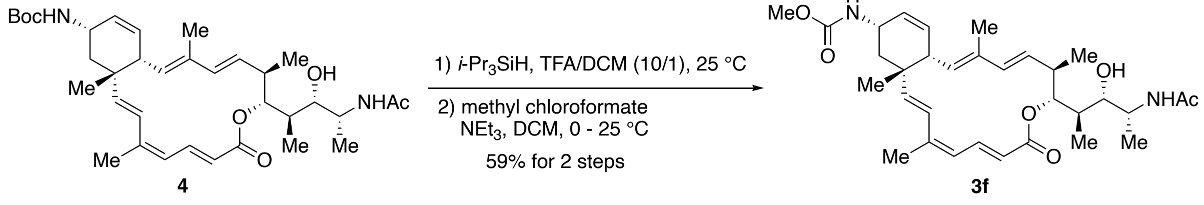

To a solution of compound **4** (4.0 mg, 0.0067 mmol) in DCM (1.0 mL) and TFA (0.1 mL) was added *i*-Pr_3SiH (4.2 µL, 0.0201 mmol) at room temperature. The resulting mixture was stirred until no starting material was observed. The solvent was removed through filtration under slow nitrogen flow. The crude solid was washed with ethyl ether and dried *in vacuo* to give compound **3a** as white powder, which was

used for the next step without further purification. To a solution of compound **3a** in DCM (0.5 ml) at 0 ℃ was added methyl chloroformate (0.8 μL, 0.010 mmol) and NEt_3 (1.9 μL, 0.0134 mmol). The reaction mixture was allowed to warm to room temperature and stirred until the reaction was complete. The mixture was concentrated *in vacuo*. Purification on silica gel column (DCM/MeOH = 50:1 to 15:1) provided compound **3f** (2.2 mg, 59% for two steps) as a white solid.

$[\alpha]^{19}_{D}$ = +158.4 (*c* 0.11, $CHCl_3$); ^1H NMR ($CDCl_3$, 300 MHz): δ 7.13 (dd, *J* = 15.2, 11.0 Hz, 1H), 6.60 (d, *J* = 16.4 Hz, 1H), 6.29 – 6.18 (m, 2H), 5.92 (d, *J* = 11.3 Hz, 1H), 5.70 (d, *J* = 15.3 Hz, 1H), 5.64 – 5.50 (m, 3H), 5.41 – 5.25 (m, 2H), 4.80 (d, *J* = 10.5 Hz, 1H), 4.67 – 4.50 (m, 2H), 4.41 – 4.25 (m, 1H), 4.25 – 4.07 (m, 2H), 3.68 (s, 3H), 3.18 – 3.09 (m, 1H), 3.03 – 2.94 (m, 1H), 2.747 – 2.63 (m, 1H), 2.39 – 2.26 (m, 2H), 1.96 (s, 3H), 1.88 (s, 3H), 1.77 (s, 3H), 1.25 (s, 3H), 1.17 (s, 3H), 1.06 (t, *J* = 6.4 Hz, 3H), 0.87 (d, *J* = 6.7 Hz, 3H); HRMS (ESI, m/z) calcd for $C_{32}H_{47}N_2O_6^+$ ($M+H^+$): 555.3434, found 555.3432.

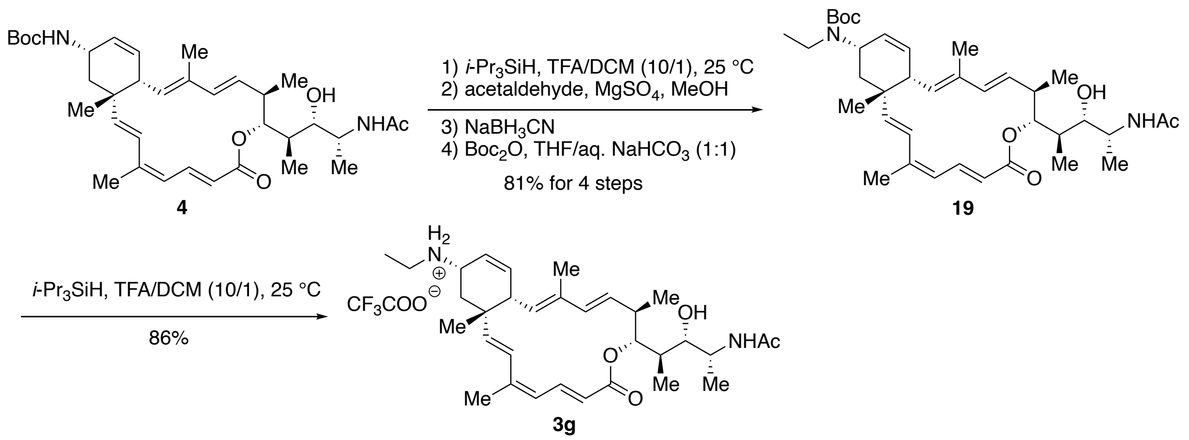

To a solution of compound **4** (2.0 mg, 0.0034 mmol) in DCM (1.0 mL) and TFA (0.1 mL) was added *i*-Pr_3SiH (2.1 μL, 0.010 mmol) at room temperature. The resulting mixture was stirred until no starting material was observed. The solvent was removed through filtration under slow nitrogen flow. The crude solid was washed with ethyl ether and dried *in vacuo* to give compound **3a** as white powder, which was used for the next step without further purification. To a solution of compound **3a** in MeOH (0.5 ml) at 0 ℃ was added acetaldehyde (0.19 μL, 0.0034 mmol) and $MgSO_4$ (1.6 mg, 0.0134 mmol). The reaction mixture was allowed to warm to room temperature and stirred for 8h, and then $NaBH_3CN$ (0.2 mg, 0.0034 mmol) was added. The reaction mixture was stirred for overnight, and diluted with H_2O and extracted with DCM. Then the combined organic extracts were washed successively with H_2O and brine, dried over anhydrous Na_2SO_4, filtered and concentrated *in vacuo* for the next step. To a solution of crude product from the previous step in THF/aq $NaHCO_3$ (0.25 mL/ 0.25 mL) was added Boc_2O (1.5 mg, 0.0067 mmol) at room temperature. The reaction mixture was stirred until the reaction was complete. Then the reaction mixture was extracted with DCM, and the combined organic extracts were washed with brine, dried over anhydrous Na_2SO_4, filtered and concentrated *in vacuo*. Purification on silica gel column (DCM/MeOH = 50:1 to 10:1) provided compound **19** (1.7 mg, 81% for four steps) as an oil.

To a solution of compound **19** (3.6 mg, 0.0058 mmol) in DCM (1.0 mL) and TFA (0.1 mL) was added *i*-Pr_3SiH (3.6 µL, 0.0173 mmol) at room temperature. The resulting mixture was stirred until no starting material was observed. The solvent was removed through filtration under slow nitrogen flow. The crude solid was washed with ethyl ether and dried *in vacuo* to provide TFA salt form of compound **3g** (1.5 mg, 86%) as a white solid.

$[\alpha]^{19}_D$ = +68.9 (*c* 0.075, $CHCl_3$); ^1H NMR ($CDCl_3$, 300 MHz): δ 9.44 (brs, 2H), 7.13 (dd, *J* = 15.1, 11.0 Hz, 1H), 6.68 (d, *J* = 16.6 Hz, 1H), 6.35 – 6.22 (m, 2H), 5.94 (d, *J* = 10.9 Hz, 1H), 5.87 – 5.77 (m, 1H), 5.77 – 5.65 (m, 1H), 5.71 (d, *J* = 14.8 Hz, 1H), 5.58 (d, *J* = 16.5 Hz, 1H), 5.41 (d, *J* = 9.9 Hz, 1H), 5.37 – 5.22 (m, 1H), 4.80 (d, *J* = 10.8 Hz, 1H), 4.25 – 4.11 (m, 1H), 3.87 – 3.73 (m, 1H), 3.20 – 2.97 (m, 4H), 2.80 – 2.65 (m, 1H), 2.09 – 1.98 (m, 2H), 1.99 (s, 3H), 1.88 (s, 3H), 1.74 (s, 3H), 1.38 (t, *J* = 7.2 Hz, 3H), 1.27 – 1.17 (m, 2H), 1.14 (s, 3H), 1.11 – 0.98 (m, 3H), 1.06 (dd, *J* = 9.3, 6.7 Hz, 3H), 0.88 (d, *J* = 6.7 Hz, 3H); HRMS (ESI, m/z) calcd for $C_{32}H_{49}N_2O_4^+$ (M+H$^+$): 525.3692, found 525.3700.

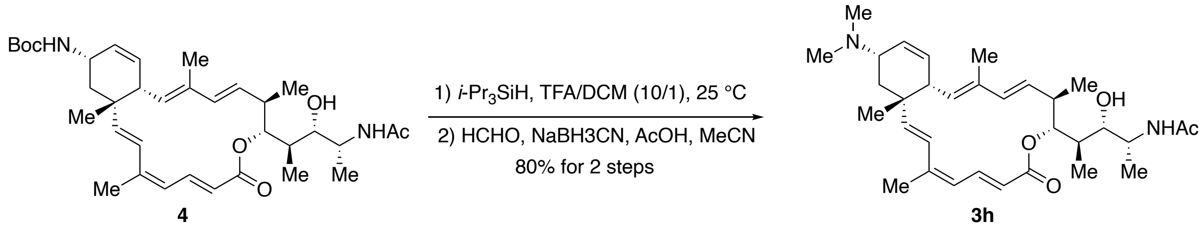

4 **3h**

To a solution of compound **4** (4.0 mg, 0.0067 mmol) in DCM (1.0 mL) and TFA (0.1 mL) was added *i*-Pr_3SiH (4.2 µL, 0.0201 mmol) at room temperature. The resulting mixture was stirred until no starting material was observed. The solvent was removed through filtration under slow nitrogen flow. The crude solid was washed with ethyl ether and dried *in vacuo* to give compound **3a** as white powder, which was used for the next step without further purification. To a solution of compound **3a** in MeOH (1.0 ml) at 0 ℃ was added formaldehyde solution (5.0 µL, 0.067 mmol, 37 wt. % in H_2O) and a catalytic amount of acetic acid. The reaction mixture was allowed to warm to room temperature and stirred for 8 h. Then $NaBH_3CN$ (4.2 mg, 0.0067 mmol) was added. The mixture was stirred for overnight, then diluted with H_2O and extracted with DCM. The combined organic extracts were washed successively with H_2O and brine, dried over anhydrous Na_2SO_4, filtered and concentrated *in vacuo*. Purification on silica gel column (DCM/MeOH = 50:1 to 10:1) provided compound **3h** (2.8 mg, 80% for two steps) as a white solid.

$[\alpha]^{19}_D$ = +82.1 (*c* 0.056, $CHCl_3$); ^1H NMR ($CDCl_3$, 300 MHz): δ 7.12 (dd, *J* = 15.2, 11.0 Hz, 1H), 6.71 (d, *J* = 16.4 Hz, 1H), 6.27 (d, *J* = 15.2 Hz, 1H), 6.20 (d, *J* = 9.0 Hz, 1H), 6.03 – 5.89 (m, 2H), 5.78 – 5.54 (m, 3H), 5.38 (dd, *J* = 15.2, 9.6 Hz, 1H), 5.28 (d, *J* = 10.0 Hz, 1H), 4.82 (d, *J* = 10.5 Hz, 1H), 4.52 (brs, 1H), 4.26 – 4.11 (m, 1H), 4.00 – 3.88 (m, 1H), 3.77 – 3.71 (m, 1H), 3.71 – 3.65 (m, 1H), 3.65 – 3.57 (m, 1H), 3.57 – 3.47 (m, 1H), 3.20 – 3.06 (m, 3H), 2.77 (s, 6H), 1.96 (s, 3H), 1.90 (s, 3H), 1.87 – 1.81 (m, 2H), 1.80 (s, 3H), 1.17 (s, 3H), 1.06 (dd, *J* = 6.8, 3.4 Hz, 3H), 0.87 (d, *J* = 6.9 Hz, 3H); HRMS (ESI, m/z) calcd for $C_{32}H_{49}N_2O_4^+$ (M+H$^+$): 525.3692, found 525.3697.

Alamar assay protocol

Alamar blue viability assays were performed using resazurin sodium salt (Sigma; St. Louis, MO) in 96 well plates. Briefly, cells were seeded in 190 uL of cell culture media at a density of 1000 cells/well and allowed to attach for 24h. Serial dilutions of biotinylated ZJ-101 were prepared at 200x final concentration in DMSO (starting from 1 mM) before being diluted 10-fold in culture media, and 10 uL of diluted drug was added to the cells for a final DMSO concentration of 0.5%. Each treatment condition was tested in triplicate. After incubation with drugs for 72 h, resazurin was added to each well to a final concentration of 10 µg/ml. After incubation at 37 °C for another 4-6 h away from direct light, fluorescence was measured using a BMG Fluostar Optima Microplate Reader (Ortenberg, Germany). Background fluorescence from wells with media plus resazurin only was subtracted from all measurements. The data were analyzed using Microsoft Excel (Redmond, WA) and Graphpad Prism (San Diego, California).

Abbreviations

DCM	Dichloromethane
DIAD	Diisopropyl azodicarboxylate
DMAP	4-Dimethylaminopyridine
DMF	Dimethylformamide
DMSO	Dimethyl sulfoxide
$Pd_2(dba)_3$	Tris(dibenzylideneacetone)dipalladium (0)
TBAF	tetra-*n*-butylammonium fluoride
TBS	*tert*-butyldimethylsilyl
TES	triethylsilyl
TFA	trifluoroacetic acid

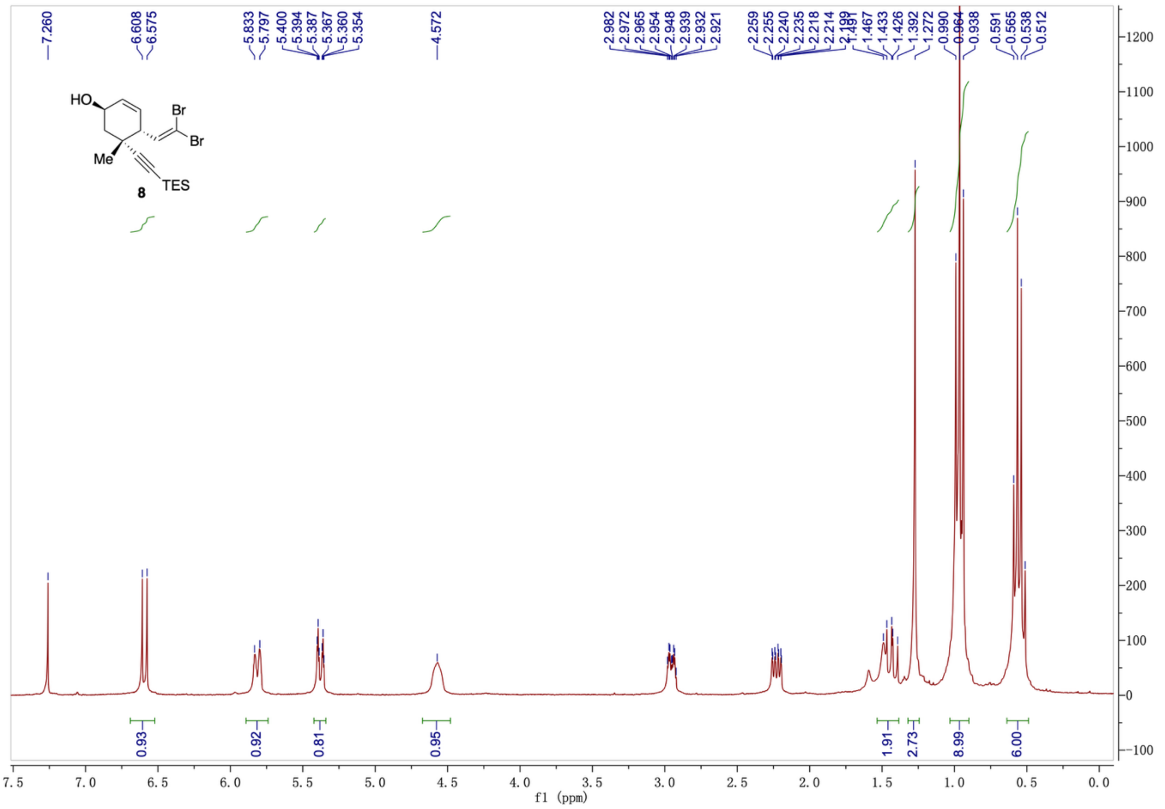
HO
Br
Br
Me
TES
8
f1 (ppm)

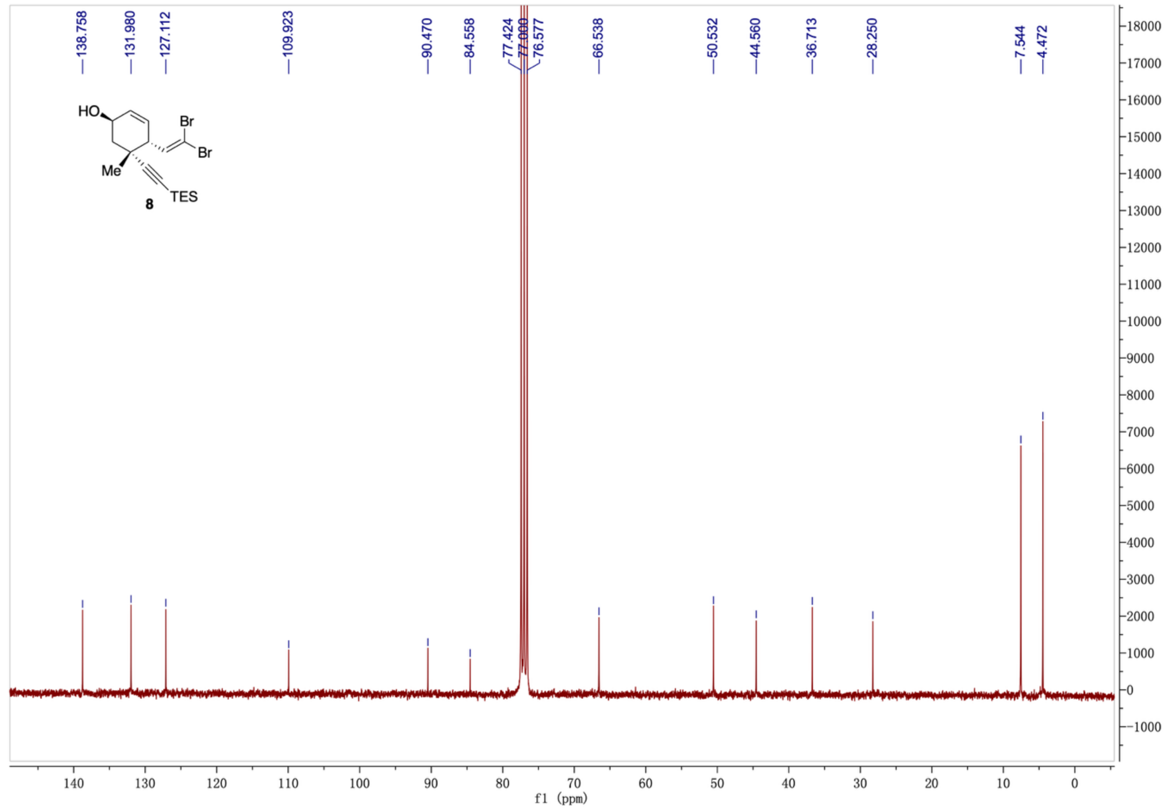
HO
Br
Br
Me
TES
8
f1 (ppm)

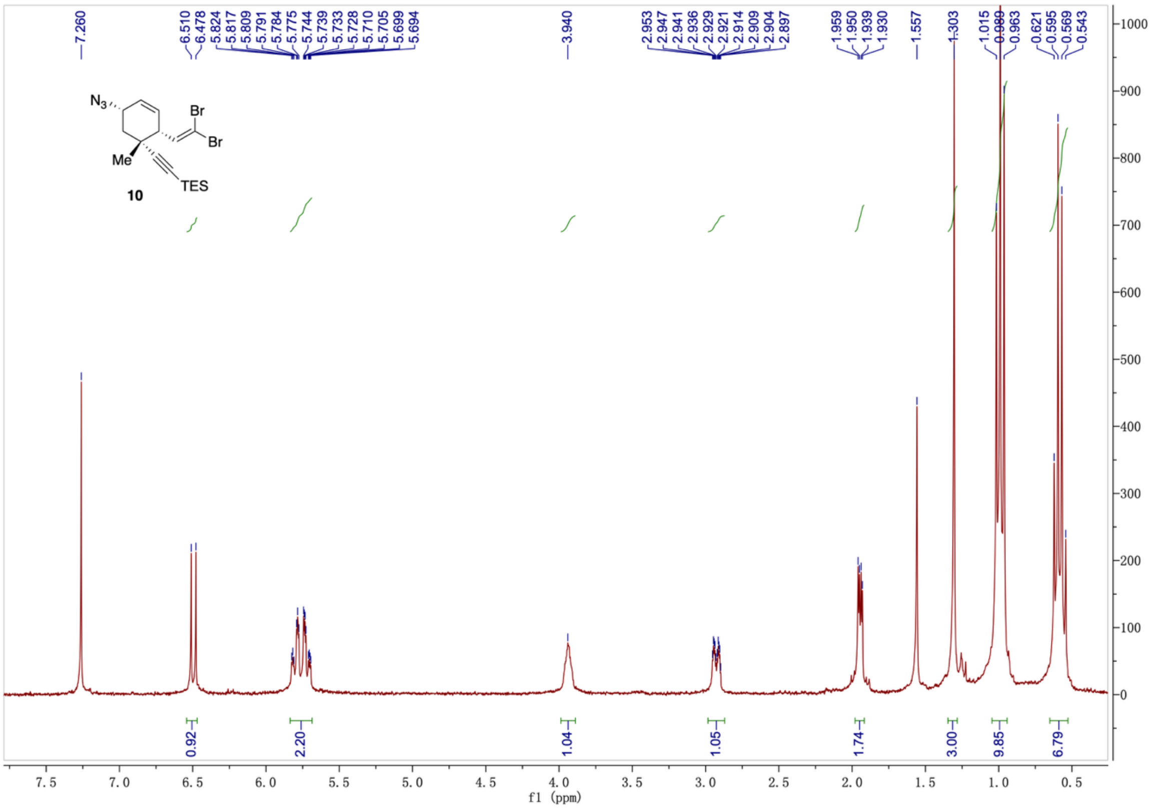

7.260
6.510
6.478
5.824
5.817
5.809
5.791
5.784
5.775
5.744
5.739
5.733
5.728
5.710
5.705
5.699
5.694
3.940
2.953
2.947
2.941
2.936
2.929
2.921
2.914
2.909
2.904
2.897
1.959
1.950
1.939
1.930
1.557
1.303
1.015
0.989
0.963
0.621
0.595
0.569
0.543
N_3
Br
Br
Me
TES
10
0.92
2.20
1.04
1.05
1.74
3.00
9.85
6.79
f1 (ppm)

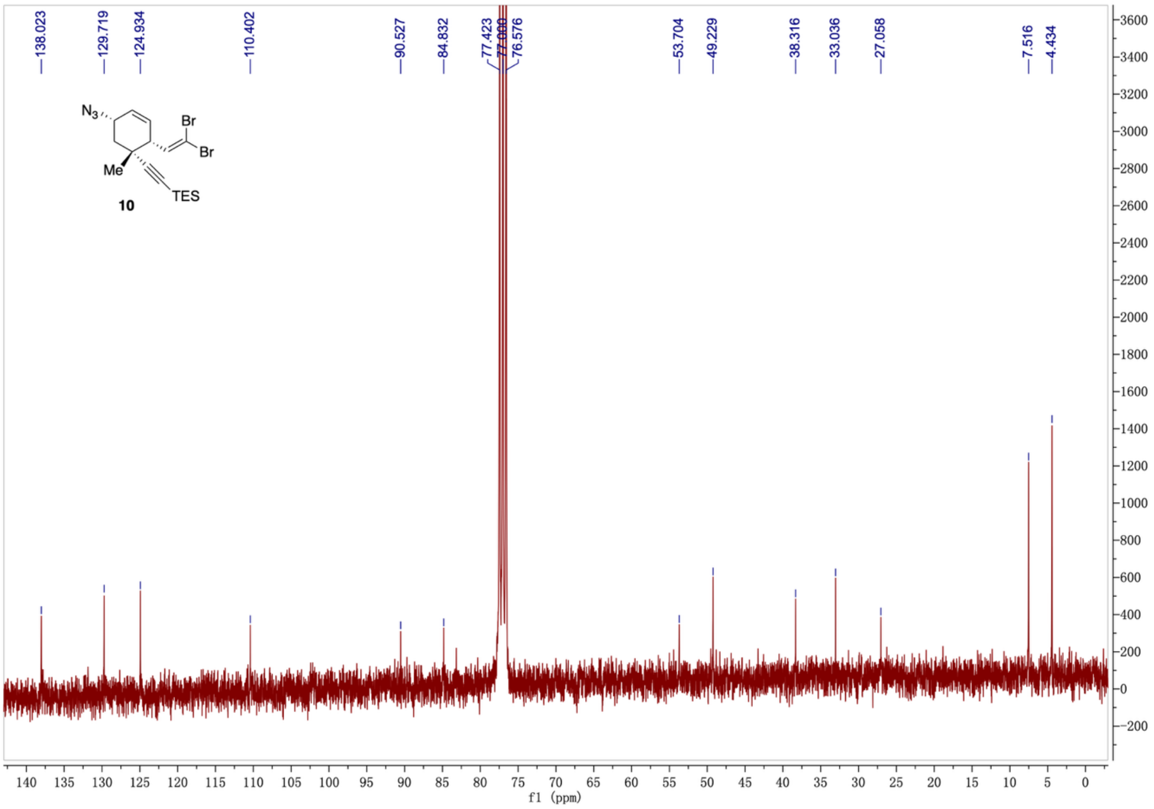

138.023
129.719
124.934
110.402
90.527
84.832
77.423
77.000
76.576
53.704
49.229
38.316
33.036
27.058
7.516
4.434
N_3
Br
Br
Me
TES
10
f1 (ppm)

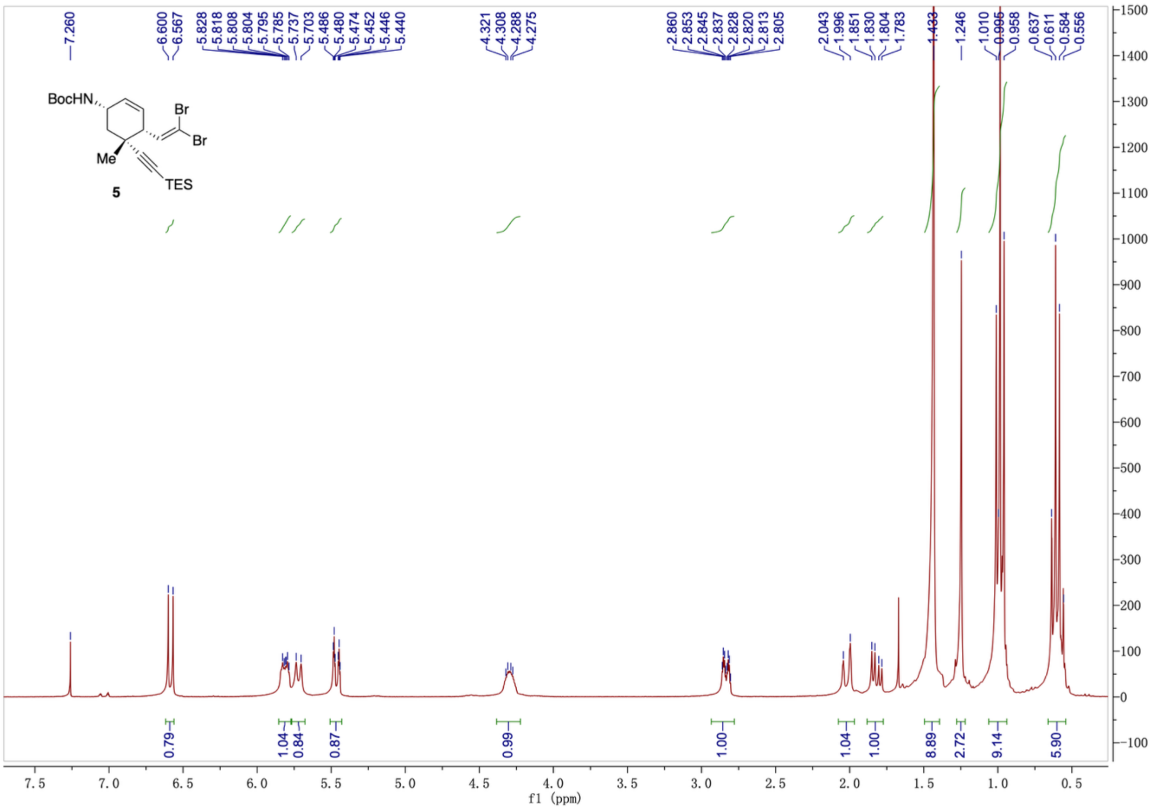
7.260
6.600
6.567
5.828
5.818
5.808
5.804
5.795
5.785
5.737
5.703
5.486
5.480
5.474
5.452
5.446
5.440
4.321
4.308
4.288
4.275
2.860
2.853
2.845
2.837
2.828
2.820
2.813
2.805
2.043
1.996
1.851
1.830
1.804
1.783
1.433
1.246
1.010
0.996
0.958
0.637
0.611
0.584
0.556
BocHN
Br
Br
Me
TES
5
0.79
1.04
0.84
0.87
0.99
1.00
1.04
1.00
8.89
2.72
9.14
5.90
f1 (ppm)

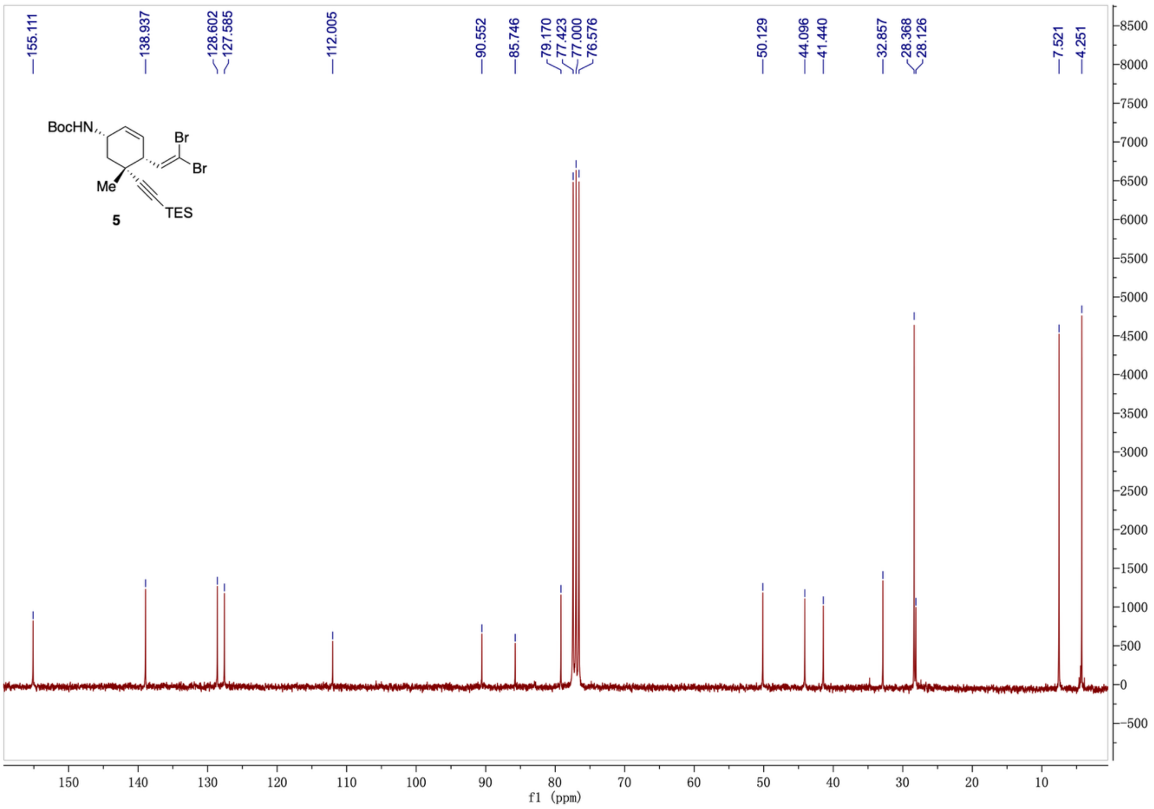
155.111
138.937
128.602
127.585
112.005
90.552
85.746
79.170
77.423
77.000
76.576
50.129
44.096
41.440
32.857
28.368
28.126
7.521
4.251
BocHN
Br
Br
Me
TES
5
f1 (ppm)

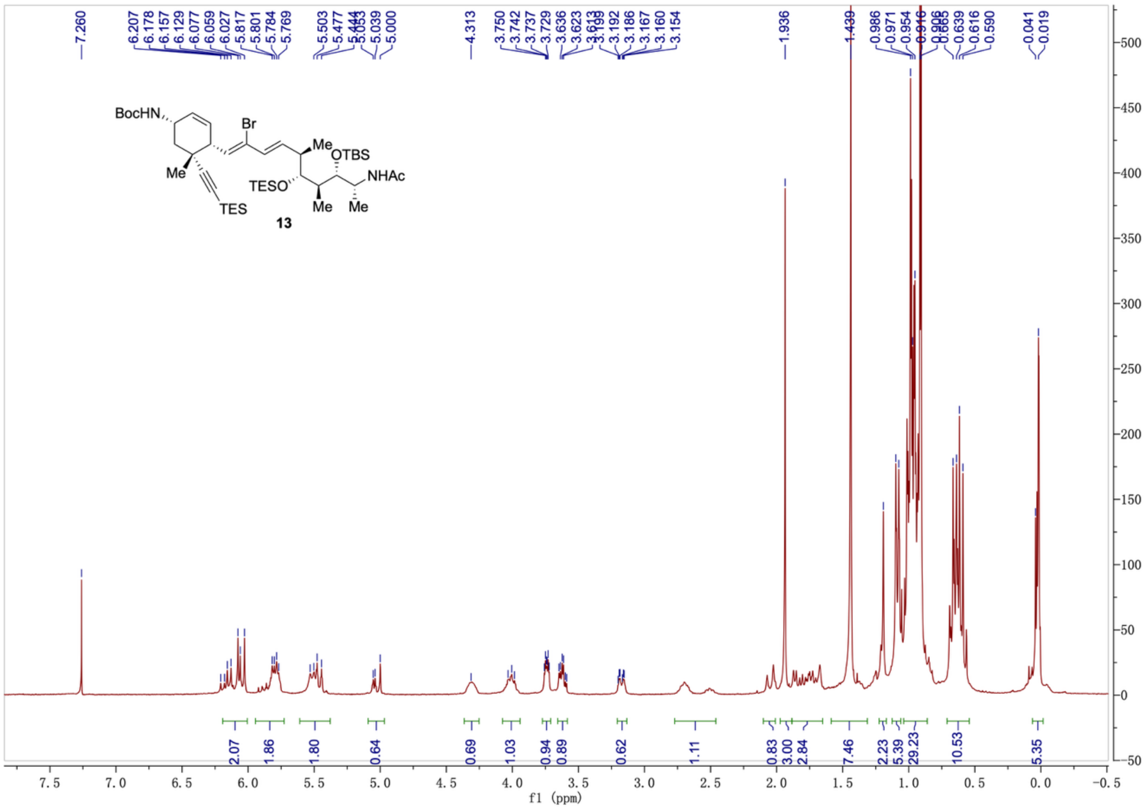
BocHN
Br
Me
OTBS
NHAc
Me
TESO
TES
Me
Me
13
f1 (ppm)

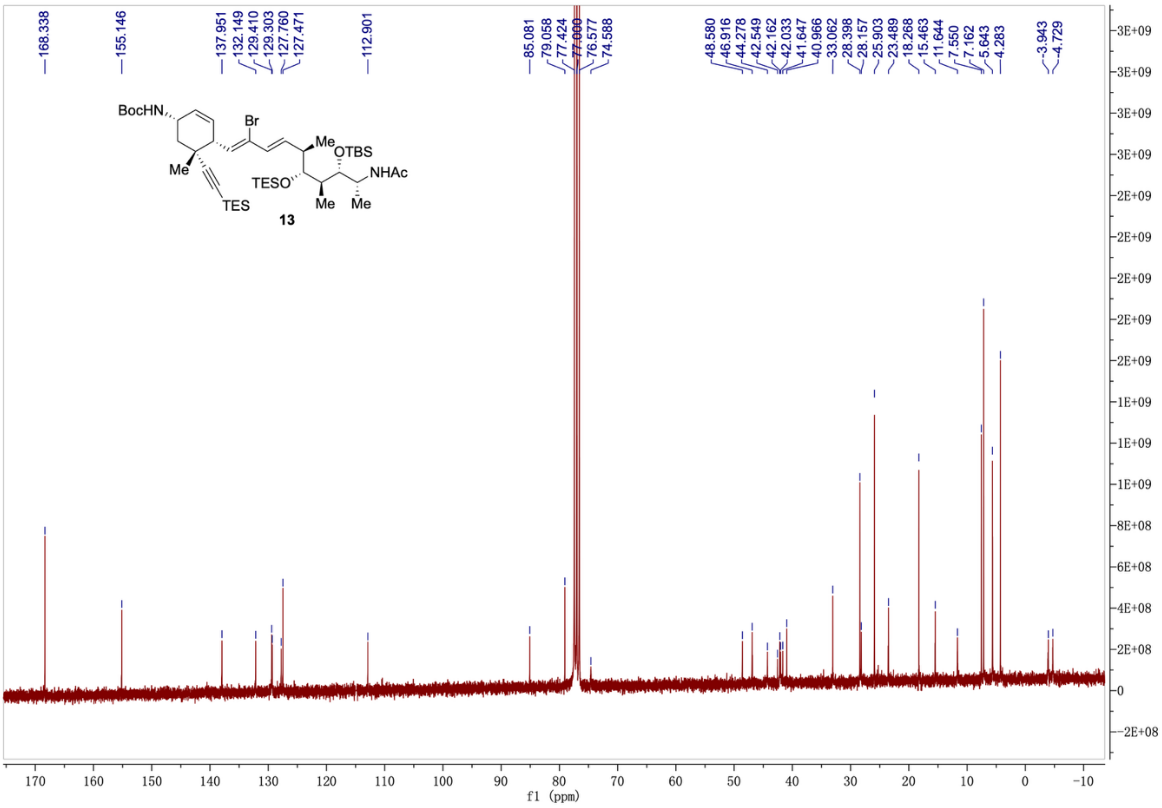
BocHN
Br
Me
OTBS
NHAc
Me
TESO
TES
Me
Me
13
f1 (ppm)

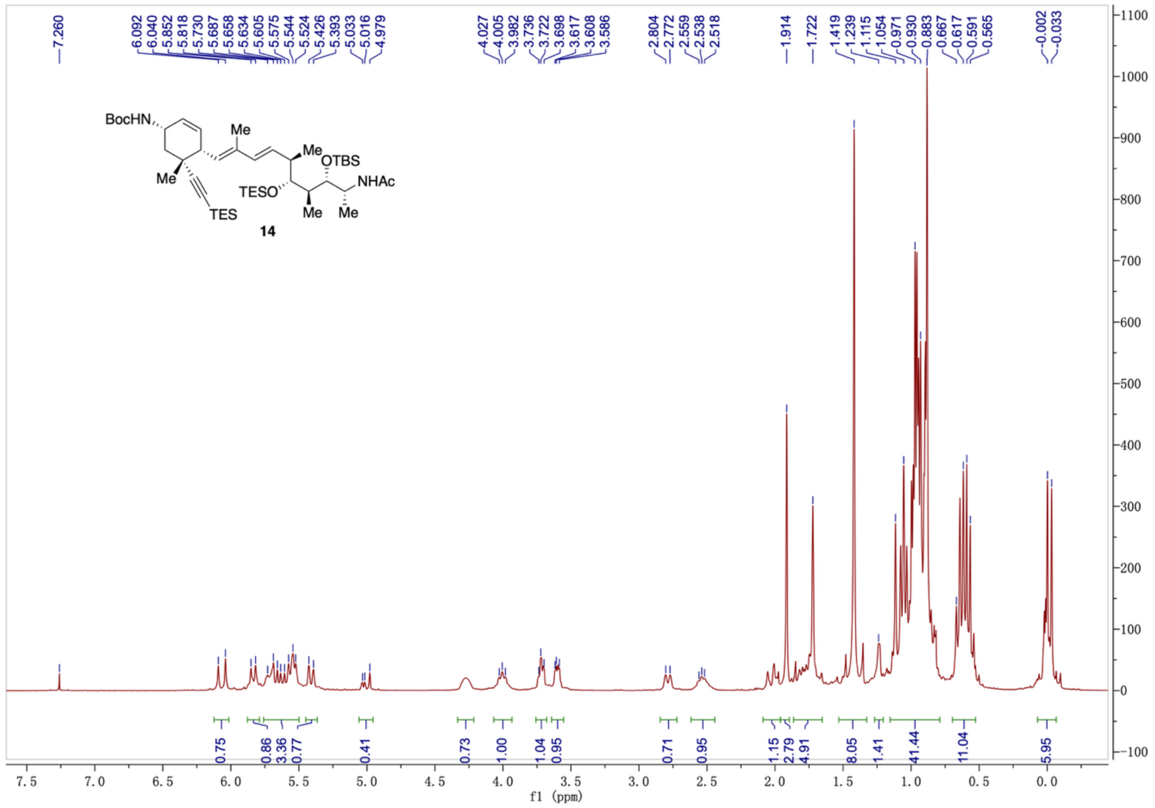

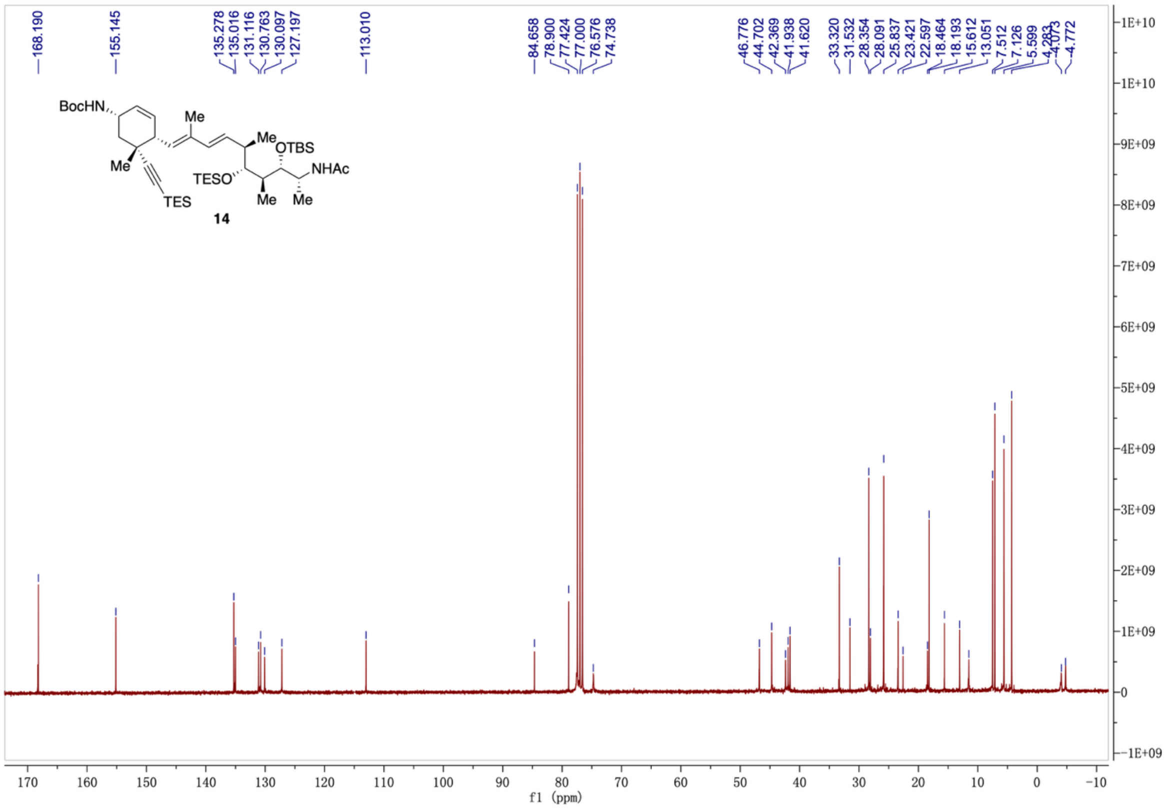

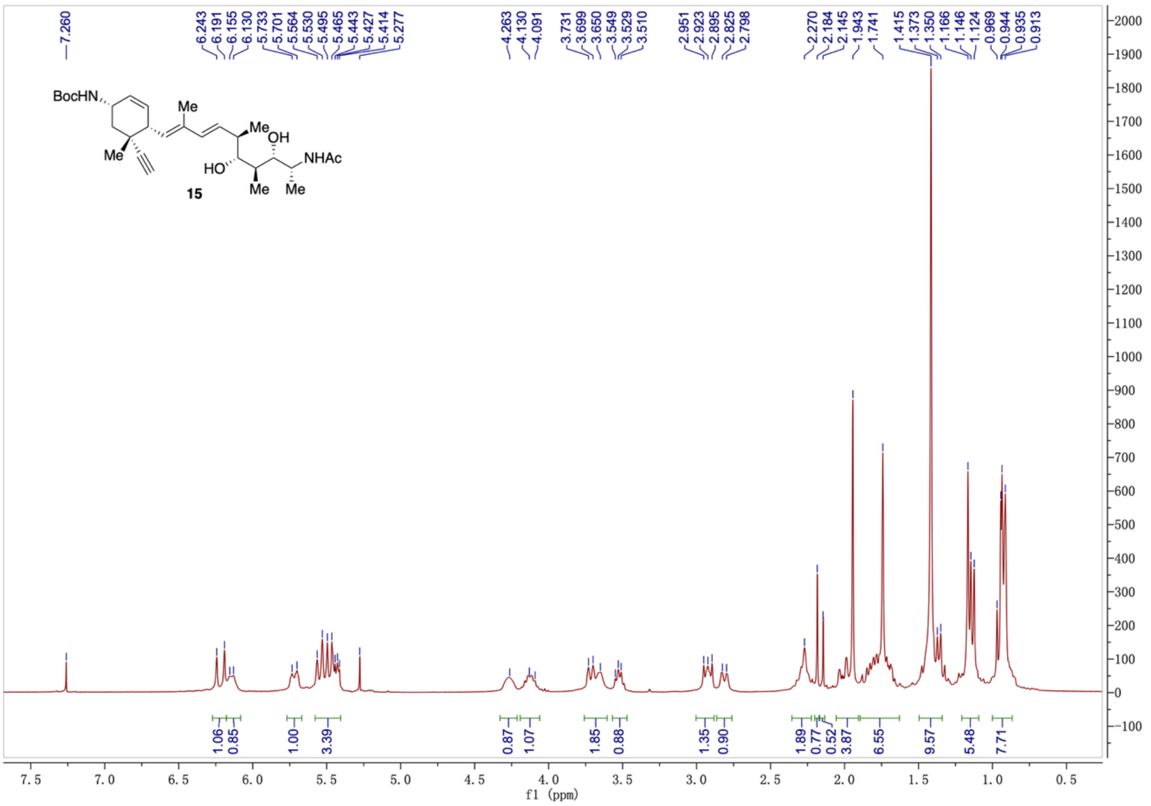

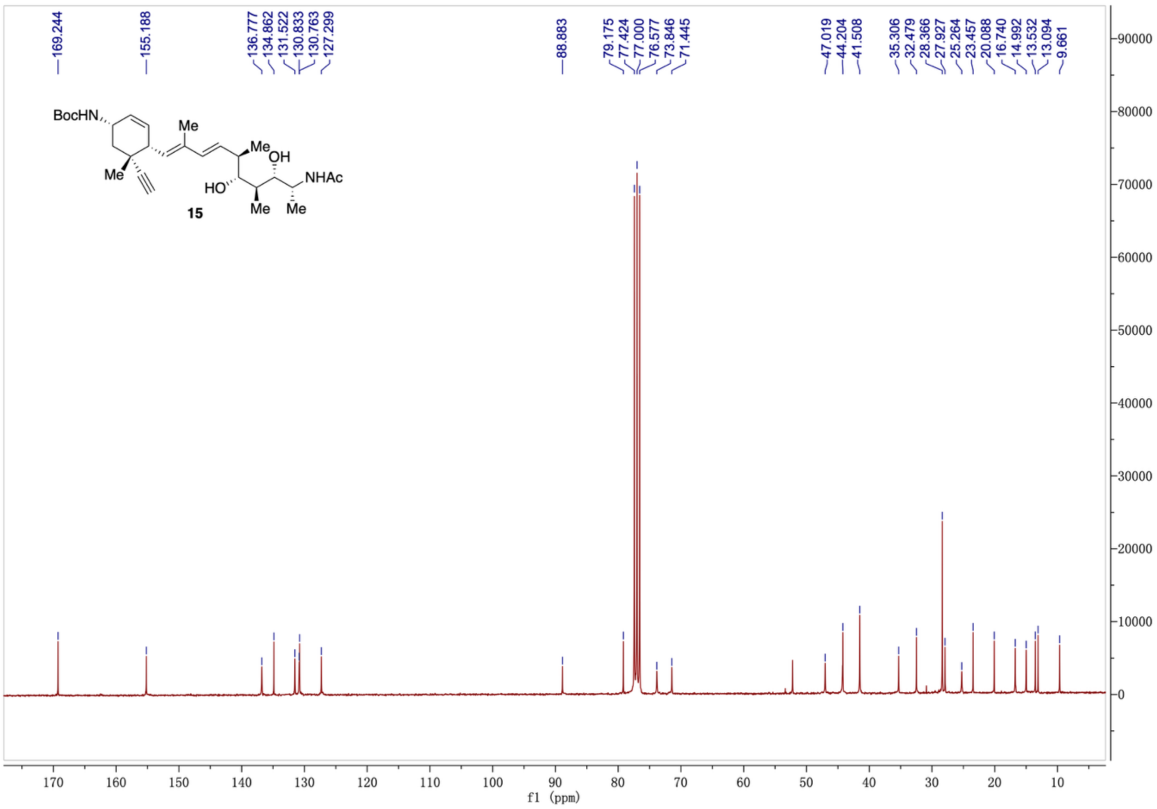

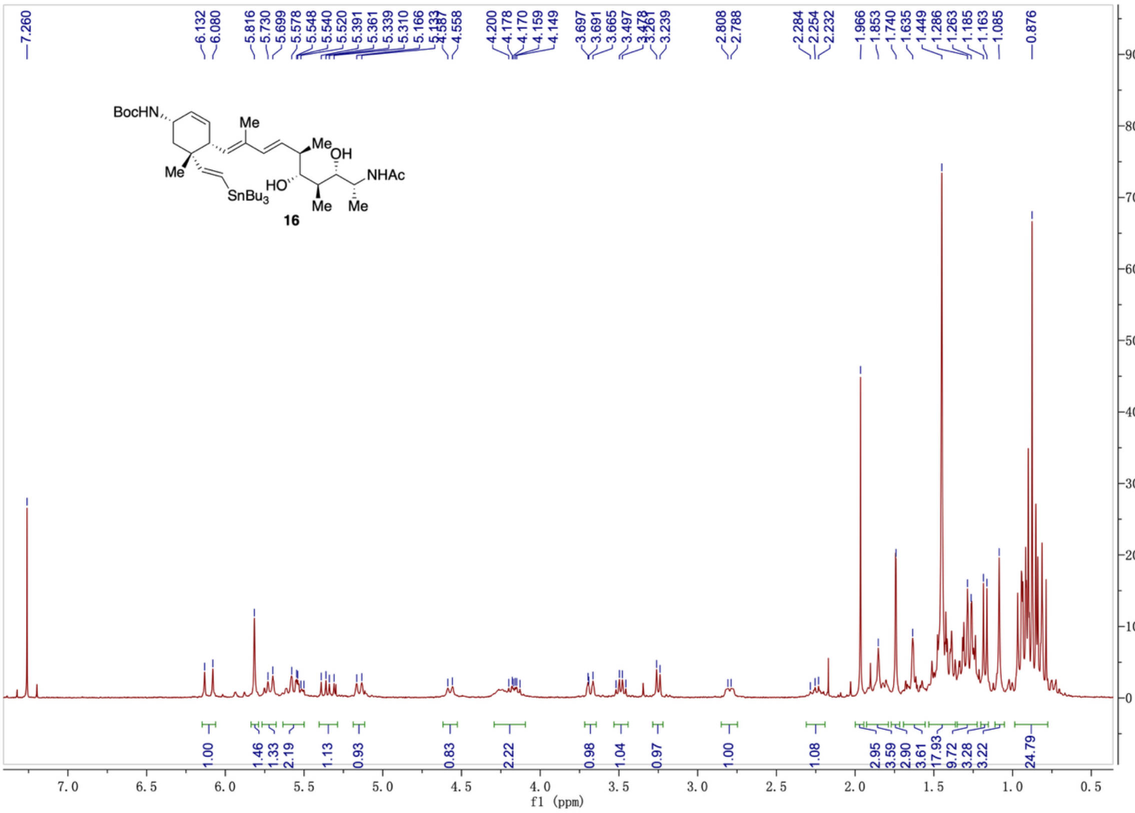

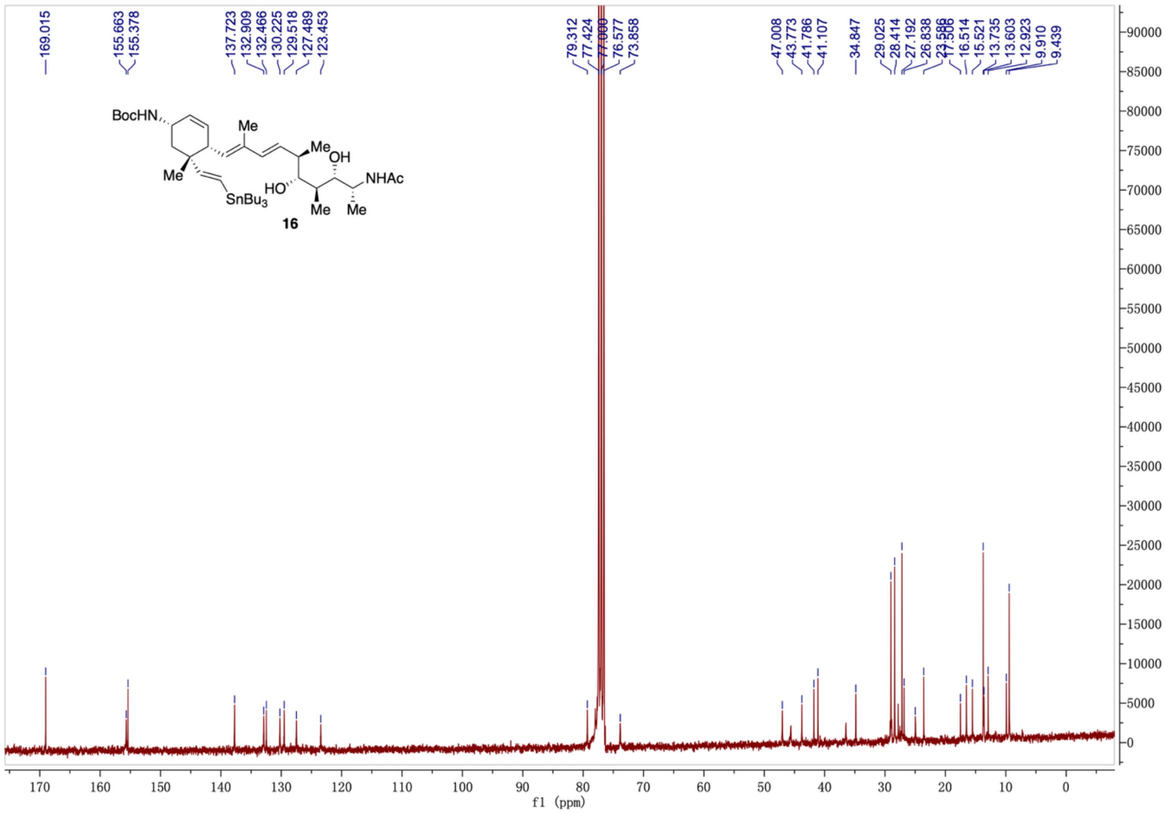

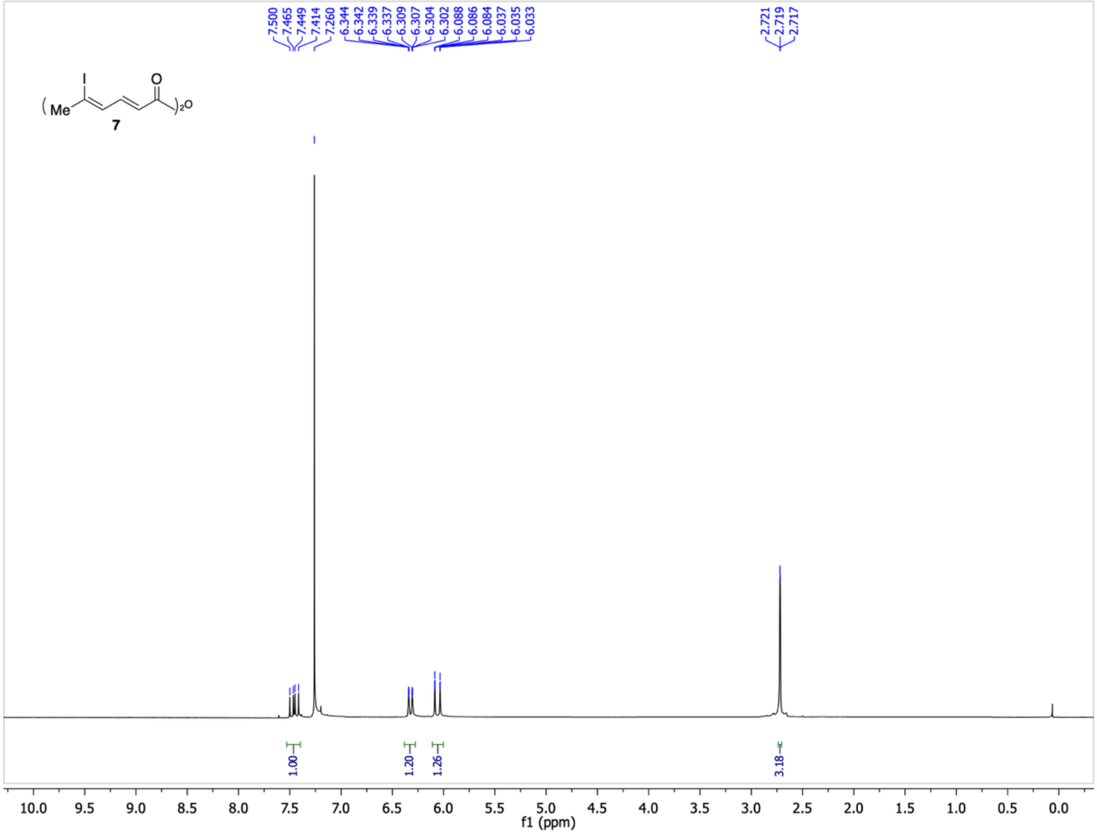

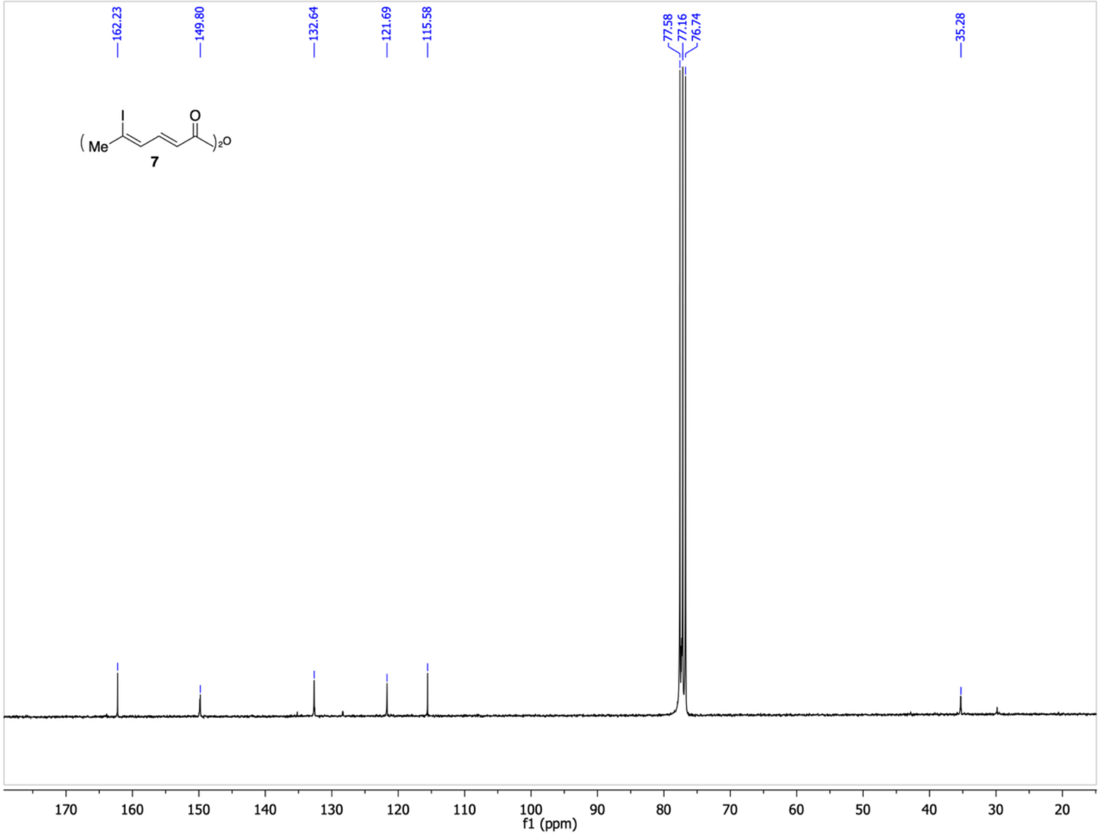

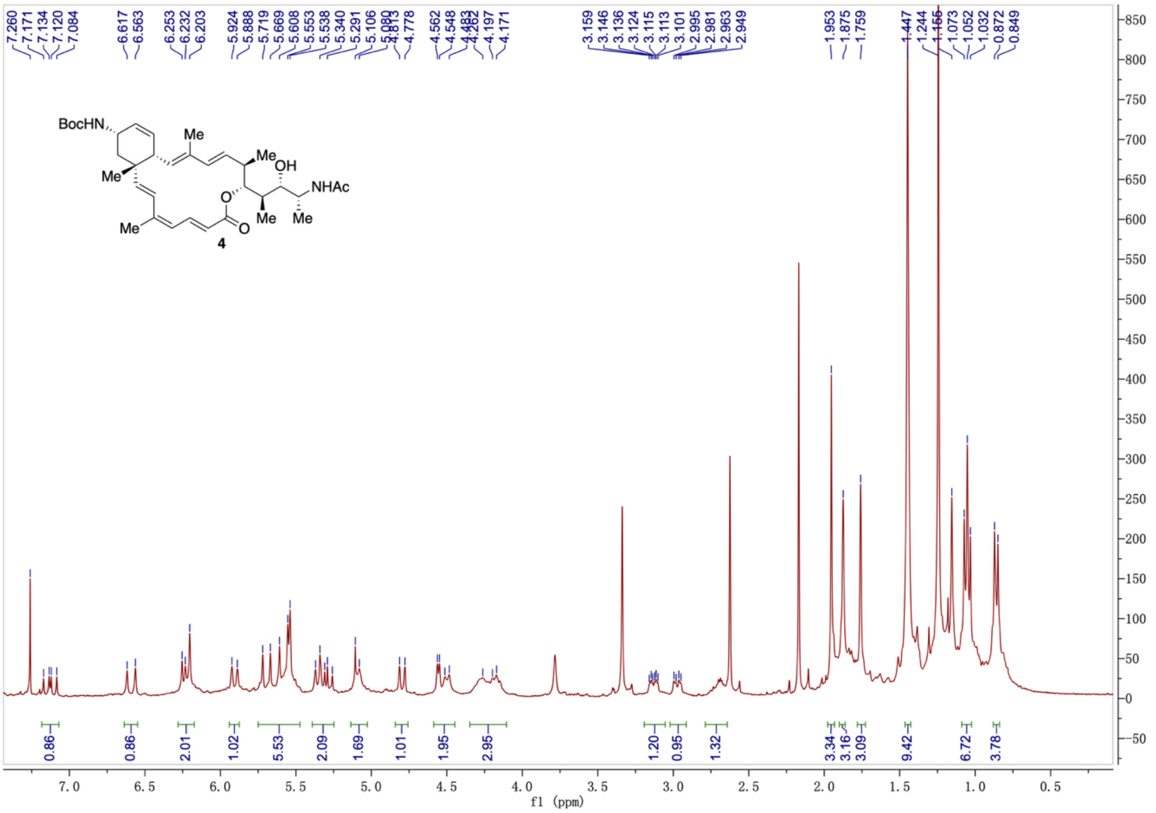

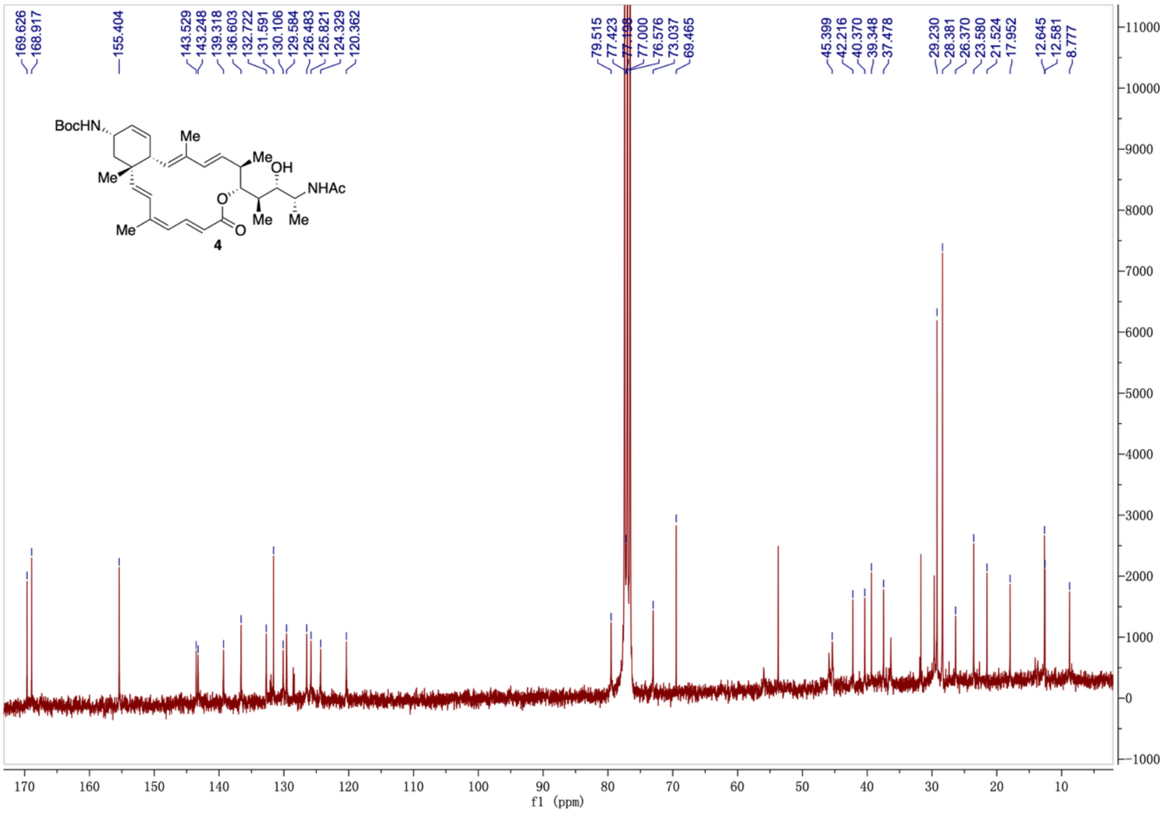

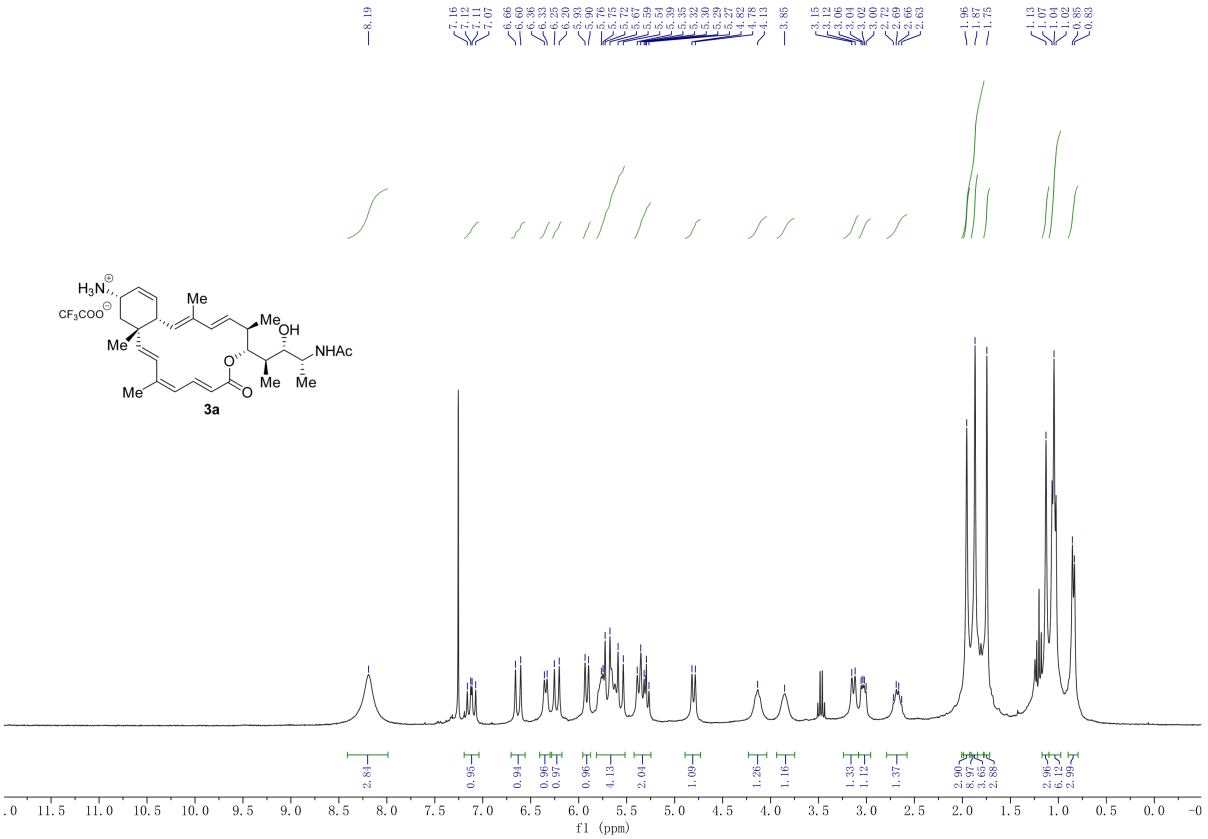
3a
f1 (ppm)

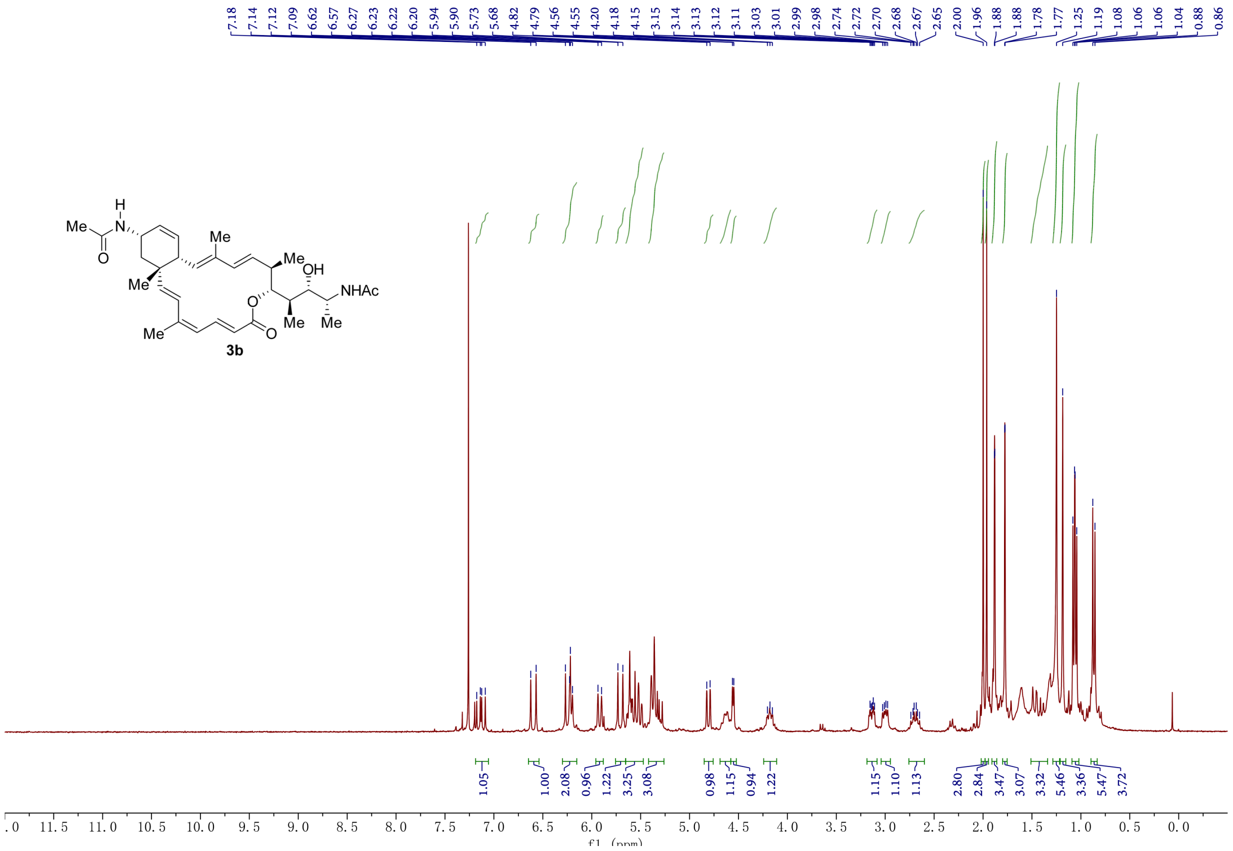
3b
f1 (ppm)

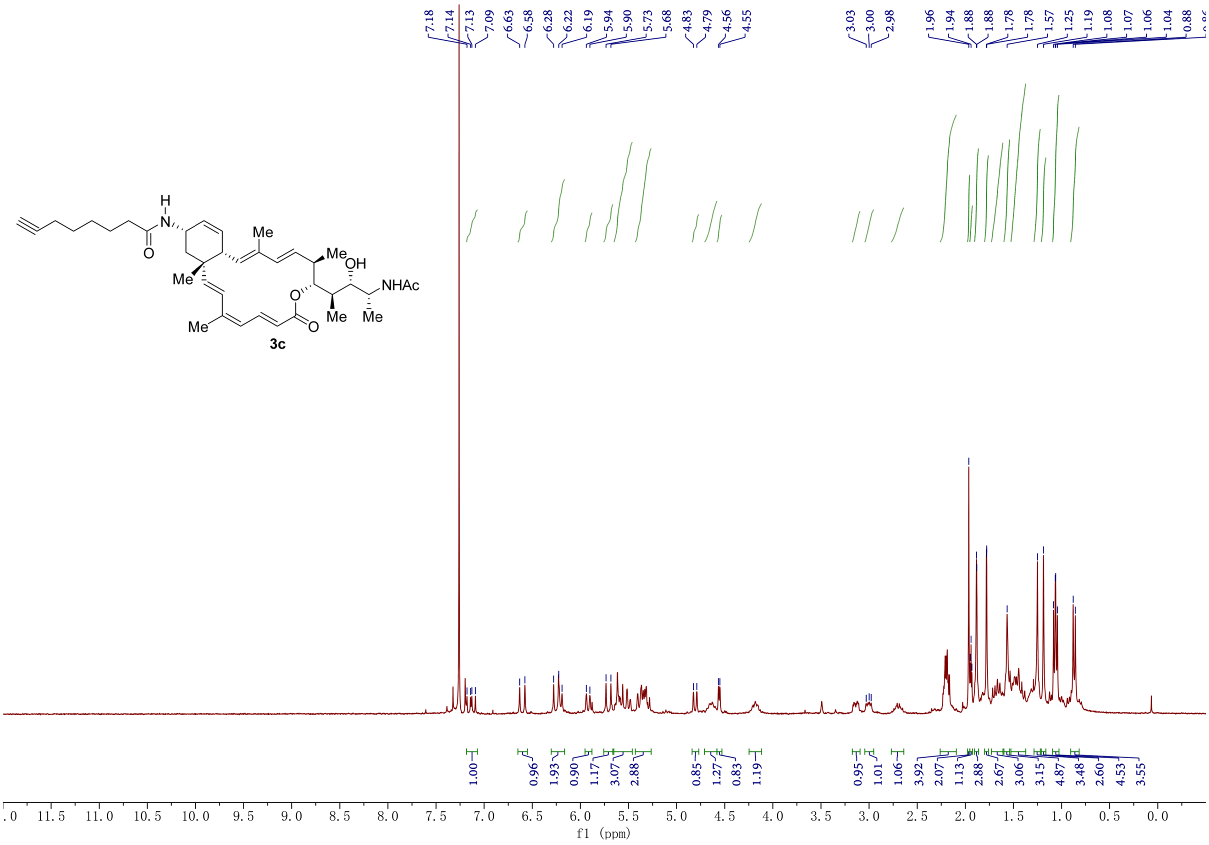

H
N
O
Me
OH
NHAc
3c
f1 (ppm)

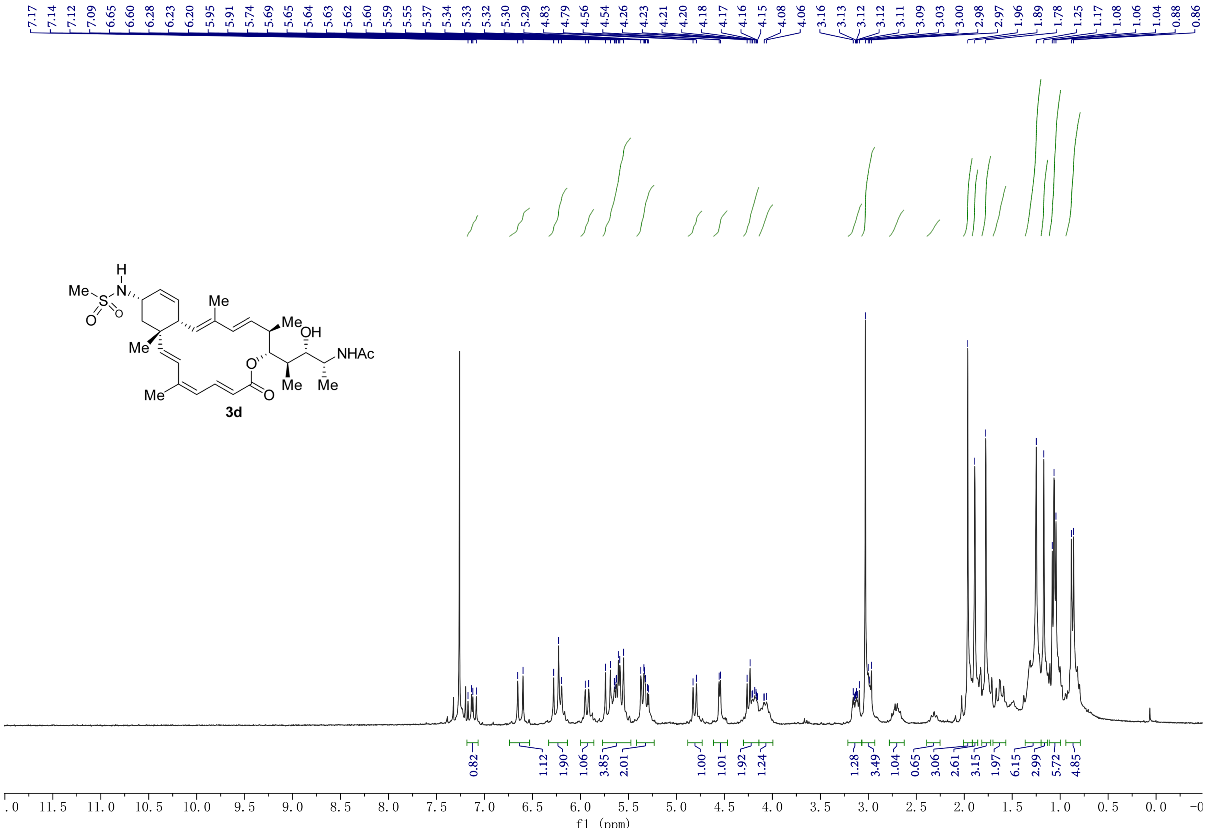

H
Me
S
N
O
OH
NHAc
3d
f1 (ppm)

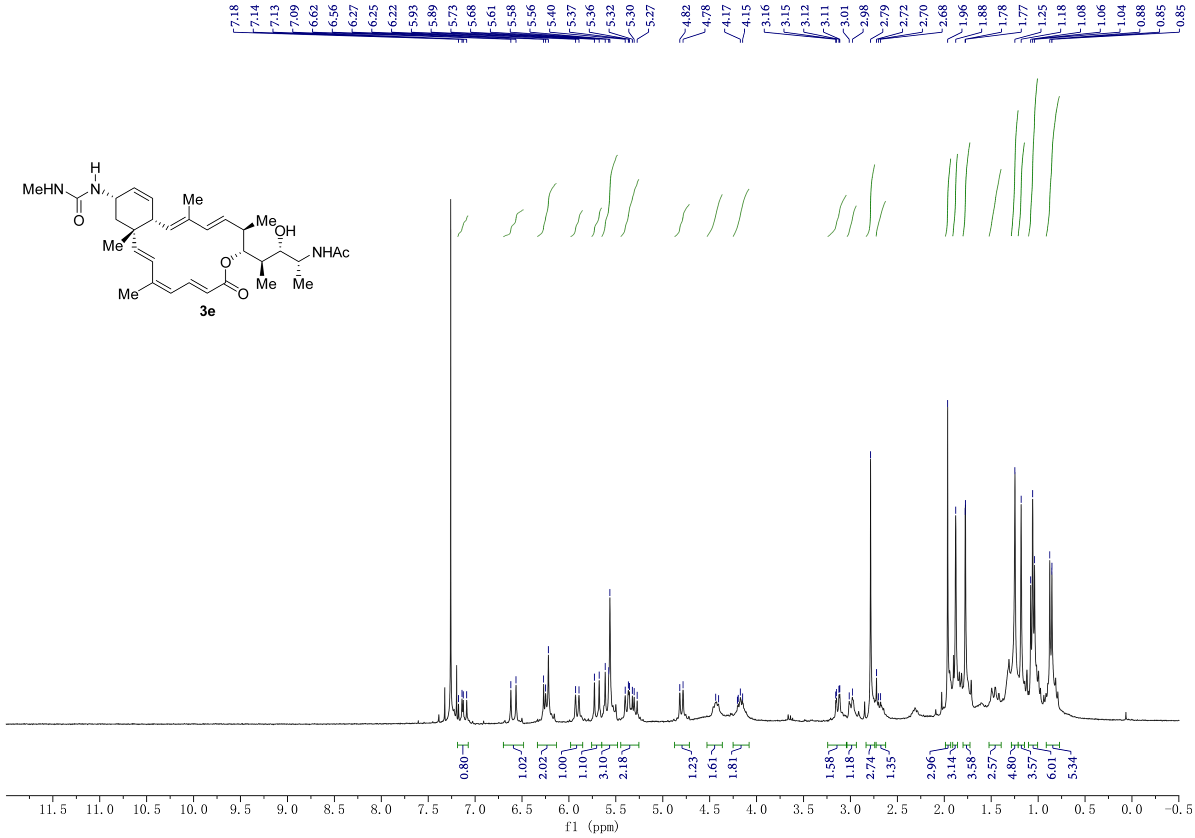
7.18 7.14 7.13 7.09 6.62 6.56 6.27 6.25 6.22 5.93 5.89 5.73 5.68 5.61 5.58 5.56 5.40 5.37 5.36 5.32 5.30 5.27 4.82 4.78 4.17 4.15 3.16 3.15 3.12 3.11 3.01 2.98 2.79 2.72 2.70 2.68 1.96 1.88 1.78 1.77 1.25 1.18 1.08 1.06 1.04 0.88 0.85 0.85
MeHN
H
N
O
Me
Me
OH
NHAc
O
Me
Me
Me
O
3e
0.80 1.02 2.02 1.00 1.10 3.10 2.18 1.23 1.61 1.81 1.58 1.18 2.74 1.35 2.96 3.14 3.58 2.57 4.80 3.57 6.01 5.34
11.5 11.0 10.5 10.0 9.5 9.0 8.5 8.0 7.5 7.0 6.5 6.0 5.5 5.0 4.5 4.0 3.5 3.0 2.5 2.0 1.5 1.0 0.5 0.0 -0.5
f1 (ppm)

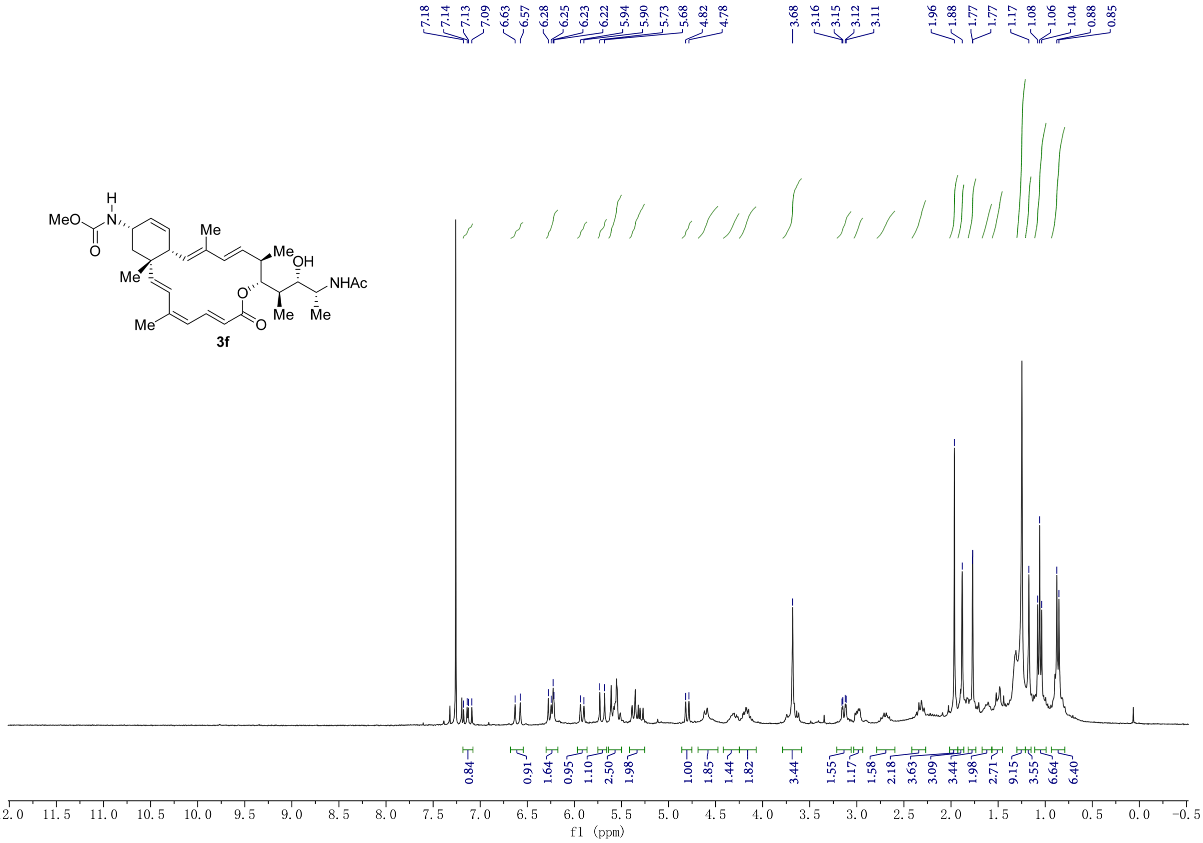
7.18 7.14 7.13 7.09 6.63 6.57 6.28 6.25 6.23 6.22 5.94 5.90 5.73 5.68 4.82 4.78 3.68 3.16 3.15 3.12 3.11 1.96 1.88 1.77 1.77 1.17 1.08 1.06 1.04 0.88 0.85
MeO
H
N
O
Me
Me
OH
NHAc
O
Me
Me
Me
O
3f
0.84 0.91 1.64 0.95 1.10 2.50 1.98 1.00 1.85 1.44 1.82 3.44 1.55 1.17 1.58 2.18 3.63 3.09 3.44 1.98 2.71 9.15 3.55 6.64 6.40
2.0 11.5 11.0 10.5 10.0 9.5 9.0 8.5 8.0 7.5 7.0 6.5 6.0 5.5 5.0 4.5 4.0 3.5 3.0 2.5 2.0 1.5 1.0 0.5 0.0 -0.5
f1 (ppm)

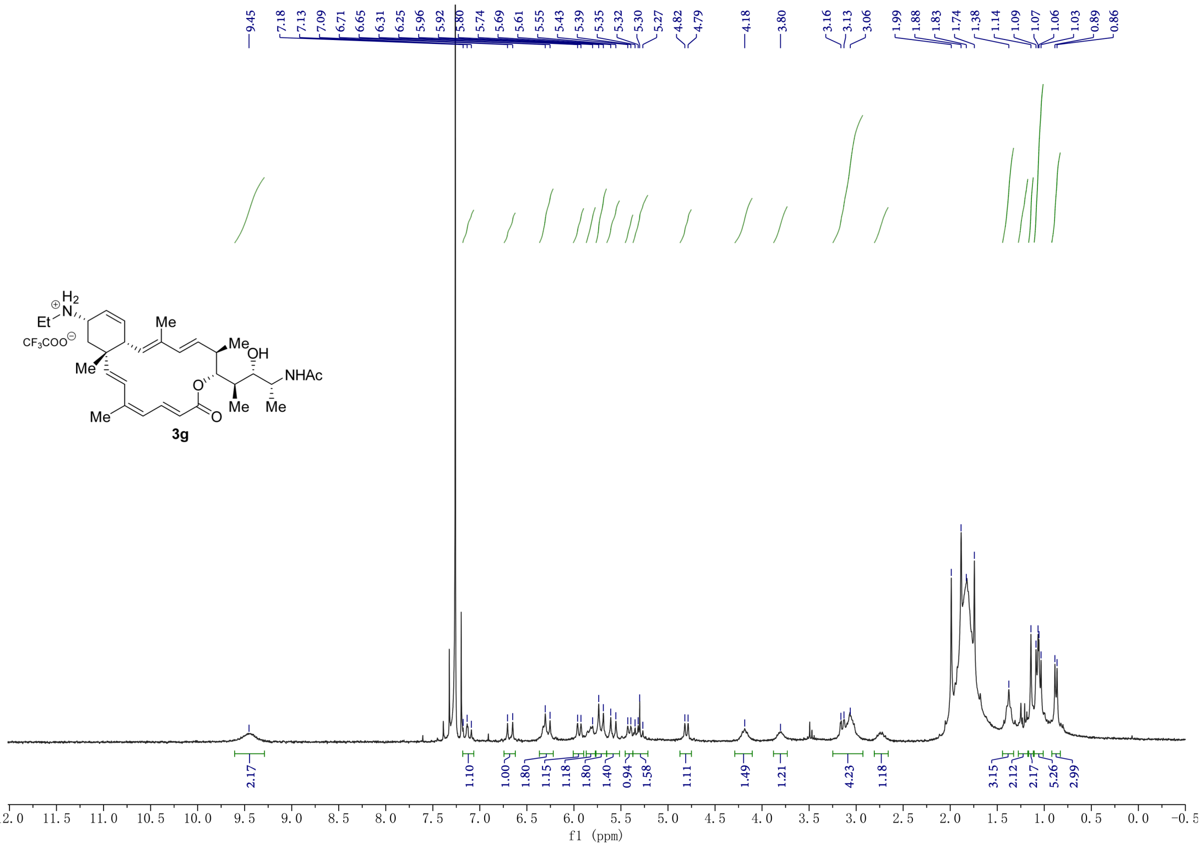
Et
CF3COO
Me
OH
NHAc
3g
f1 (ppm)

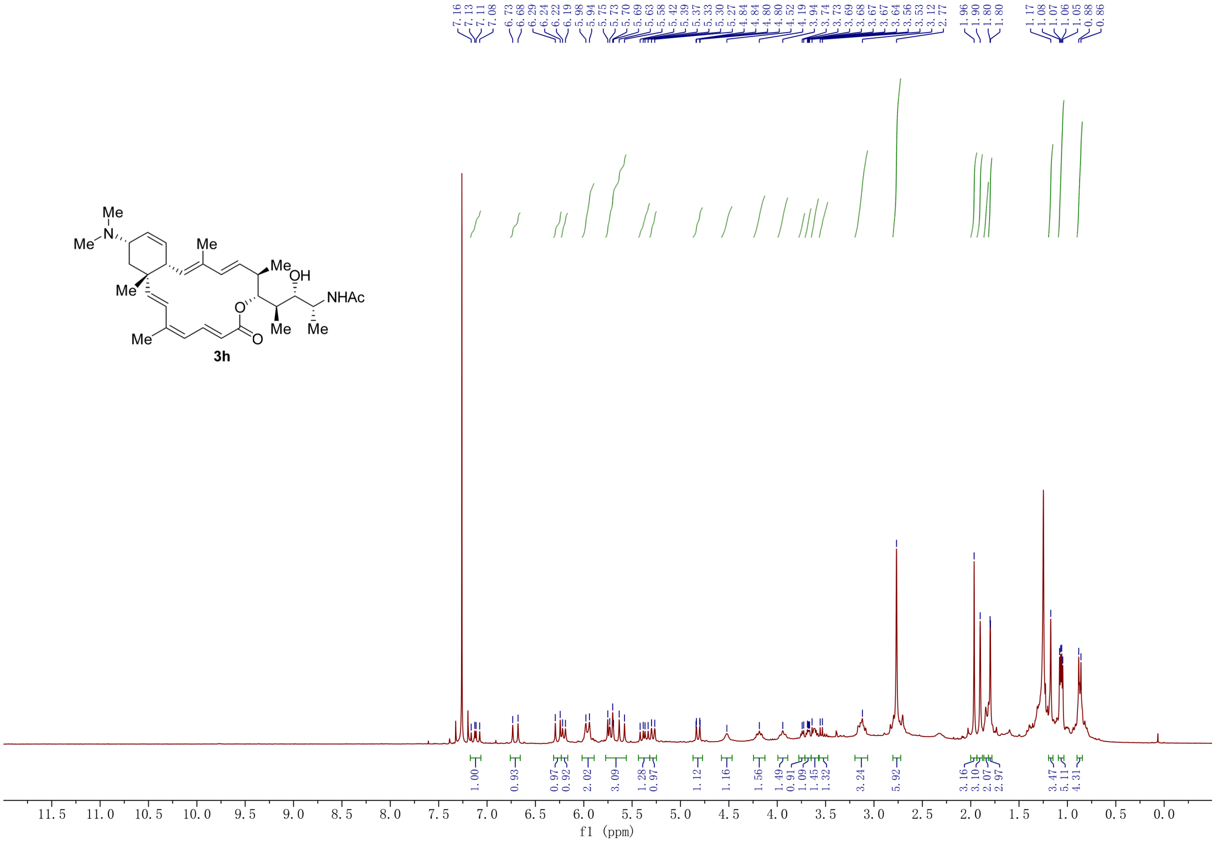
Me
OH
NHAc
3h
f1 (ppm)
